# Pelvic tilt remains unchanged after periacetabular osteotomy: A single‐arm multilevel meta‐analysis and meta‐regression

**DOI:** 10.1002/jeo2.70453

**Published:** 2025-10-15

**Authors:** Nikolai Ramadanov, Maximilian Voss, Maximilian Heinz, Robert Hable, Robert Prill, Roland Becker, Plamen Penchev, Sufian S. Ahmad

**Affiliations:** ^1^ Center of Orthopaedics and Traumatology, Brandenburg Medical School University Hospital Brandenburg an der Havel Brandenburg an der Havel Germany; ^2^ Faculty of Health Science Brandenburg Brandenburg Medical School Theodor Fontane Brandenburg an der Havel Germany; ^3^ Faculty of Applied Computer Science Deggendorf Institute of Technology Deggendorf Germany; ^4^ Faculty of Medicine Medical University of Plovdiv Plovdiv Bulgaria; ^5^ Department of Orthopaedic Surgery Hannover Medical School Hannover Germany

**Keywords:** dysplasia, meta‐analysis, pelvic tilt, periacetabular osteotomy, systematic review

## Abstract

**Purpose:**

Understanding changes in pelvic tilt (PT) after periacetabular osteotomy (PAO) is essential for surgical planning and outcome assessment. This study aimed to synthesise current evidence on pre‐ and postoperative PT and identify associated factors using meta‐regression.

**Methods:**

A systematic search of PubMed, Embase, and Epistemonikos was conducted through 31 May 2025. A single‐arm multilevel meta‐analysis was performed using a random‐effects model to estimate mean PT values before and after PAO. Subgroup analyses examined differences based on surgical indication (DDH/BDDH vs. others) and unilateral vs. bilateral surgery. Meta‐regression assessed the impact of age, sex and BMI on PT outcomes. Statistical analyses were performed using R.

**Results:**

Twelve primary studies comprising 680 patients (785 hips) were included. The mean preoperative pelvic tilt (PT) was 8.13° and decreased slightly postoperatively to 6.81°, with no statistically significant overall change. The greatest positional change was observed in the sitting position (mean difference: 12.01°), though this was also not statistically significant. Subgroup analyses showed no significant differences in PT based on operative indication or unilateral vs. bilateral surgery. Meta‐regression identified patient age as a significant predictor of postoperative PT in the sitting position (*β* = –4.45, 95% CI: –8.48 to –0.41; *p* = 0.0454), with older age associated with lower PT values. No significant associations were found for sex or BMI.

**Conclusion:**

Pelvic tilt remains largely unchanged after PAO, regardless of operative indication or surgical laterality. However, older age is associated with lower postoperative PT in the sitting position, highlighting the importance of considering individual patient characteristics in surgical planning and assessment.

**Level of Evidence:**

Level IIa, systematic review and meta‐analysis of predominantly prospective and retrospective cohort studies.

**Abbreviations**
DDH: developmental dysplasia of the hip
LCEA: lateral center‐edge angle
MCID: minimum clinically important difference
PAO: periacetabular osteotomy
PRISMA: Preferred Reporting Items for Systematic Reviews and Meta‐Analyses
PROM: patient‐reported outcome measure
PROSPERO: International Prospective Register of Systematic Reviews
PT: pelvic tilt
RCT: randomised controlled trial
RoB: Risk of bias
ROBINS‐I: Risk Of Bias In Non‐randomised Studies of Interventions

## INTRODUCTION

Developmental dysplasia of the hip (DDH) is a structural abnormality characterised by insufficient acetabular coverage of the femoral head, which can lead to joint instability, cartilage damage, and early osteoarthritis. In skeletally mature patients, periacetabular osteotomy (PAO) is the most widely used joint‐preserving surgical technique. The procedure realigns the acetabulum to improve femoral head coverage and load distribution, alleviating symptoms and preventing degenerative progression. Numerous long‐term studies support its effectiveness: survivorship of the native hip exceeds 80%–90% at 10–15 years postoperatively in appropriately selected patients, particularly those with minimal arthritis and good joint congruency [38]. By restoring biomechanical alignment, PAO can significantly delay or avoid total hip arthroplasty (THA) in young adults with DDH, offering functional improvement and pain relief [1, 20, 24]. PAO is indicated in patients with symptomatic DDH and preserved femoral head congruency, provided the femoral head remains concentrically located within the acetabulum without evidence of subluxation and with minimal to no degenerative change. A recently published multilevel meta‐analysis showed PAO provides good short‐ to mid‐term outcomes even in borderline DDH [27].

In parallel with advances in hip preservation surgery, pelvic tilt (PT) has gained increasing attention as a biomechanical parameter affecting hip joint orientation and function [16]. PT refers to the sagittal rotation of the pelvis, which alters the functional position of the acetabulum, femoral head coverage, and loading. In total hip arthroplasty, abnormal PT—particularly excessive posterior PT—has been linked to component malpositioning and dislocation risk [19]. This has led to broader awareness of spinopelvic alignment in both diagnosis and surgical planning across hip procedures.

In the context of PAO, PT is particularly relevant. It remains uncertain whether patients with DDH adopt increased anterior PT as a compensatory mechanism to enhance anterior coverage, whether minor changes in PT after PAO reflect a trend toward normalisation following improved acetabular orientation, or whether PT remains relatively stable pre‐ and postoperatively, with only small, patient‐specific variations. Nonetheless, preoperative PT can influence radiographic measurements like the lateral center‐edge angle and acetabular version, potentially affecting diagnosis and surgical decision‐making.

We hypothesised that reorientation of the acetabulum through PAO alters hip joint congruency and impingement‐free range of motion, which in turn may lead to adaptive changes in pelvic tilt in the sagittal plane, particularly during activities requiring deep hip flexion, such as sitting.

Given these biomechanical considerations, understanding whether and how PT changes after PAO is essential. This study aims to synthesise current evidence on pre‐ and postoperative PT in patients undergoing PAO through a single‐arm multilevel meta‐analysis, and to identify associated factors using meta‐regression analysis.

## METHODS

### Reporting standards and protocol registration

This study was conducted in accordance with the updated PRISMA 2020 guidelines for transparent and comprehensive reporting [25]. The corresponding PRISMA checklist is available as Supporting Information: Figure S1. Database searches commenced immediately following registration approval of the study protocol with PROSPERO (International Prospective Register of Systematic Reviews) on 12 May 2025.

### Search strategy and data sources

A comprehensive literature search was undertaken across multiple electronic databases, including PubMed, Embase, and Epistemonikos. The search covered all records up to 31 May 2025. Search terms included combinations of keywords and MeSH terms relating to PAO and PT: (((periacetabular osteotomy) OR (PAO)) AND ((pelvic tilt) OR (pelvic orientation) OR (spinopelvic alignment))). Search syntax was tailored to each platform's requirements. No language or publication date restrictions were applied.

### Study selection process

Studies were screened through a two‐step selection protocol. Titles and abstracts were independently reviewed for eligibility by two authors (NR and MV). Full‐text review followed for potentially eligible studies. Disagreements were resolved by a third reviewer (SJA). The degree of inter‐reviewer agreement was quantified using Cohen's kappa coefficient (*κ*) to ensure reliability of the selection process.

### Eligibility criteria

Eligible studies included randomised and non‐randomised studies, whether prospective or retrospective, as well as case series. Excluded materials encompass single case reports, expert opinion, editorials, and reviews. Only studies reporting preoperative and/or postoperative PT values in degrees in patients undergoing PAO were considered.

### Primary outcome definition

The primary outcome was PT, defined as the sagittal angular orientation of the pelvis, commonly derived from lateral radiographic imaging, computed tomography or 3D imaging. All recognised measurement methods were accepted, for example, manual PT measurement, statistical shape modelling, and CT segmentation. PT was reported in degrees (°), with anterior and posterior tilts represented by positive and negative values, respectively.

### Data extraction procedure

Data collection was performed independently by two reviewers (NR and MV), using a standardised form. Extracted information was encompass study characteristics (author, year, country, design), sample size, patient demographics, follow‐up duration, methods for PT measurement, pre‐ and/or postoperative PT values, and information on risk of bias. Any discrepancies were reviewed and adjudicated by a third reviewer (SJA).

### Quality assessment

Two independent reviewers (NR and MV) evaluated the methodological quality of all included studies. Non‐randomised studies were assessed using the Risk Of Bias In Non‐randomised Studies of Interventions (ROBINS‐I) tool [31]. Inconsistencies between reviewers were resolved through consensus or, if needed, by consulting a third reviewer (SJA). To assess the potential for publication bias, Begg's test and visual inspection of funnel plots were conducted.

### Data synthesis and meta‐regression analysis

A single‐arm multilevel meta‐analysis was conducted to synthesise the mean values of pre‐ and postoperative PT in patients undergoing PAO. A random‐effects model with inverse‐variance weighting was employed to account for between‐study variability. Confidence intervals were calculated using the restricted maximum likelihood heterogeneity estimator with Hartung–Knapp adjustment [26] for random‐effects models. Statistical heterogeneity was assessed using the Higgins' *I*² statistic and categorised as low (<25%), moderate (25%–75%), or high (>75%). A subgroup analysis was conducted to evaluate the potential impact of operative indication—specifically comparing patients with DDH or borderline DDH versus those with other indications—and the number of hips operated on per patient (unilateral vs. bilateral). This analysis aimed to identify or exclude significant differences in PT outcomes across these subgroups.

To identify potential influencing factors on pre‐ and postoperative PT, a meta‐regression was performed for random‐effects models. Univariate regression models were fitted for each covariate. Between‐study heterogeneity was evaluated using Cochrane's QE‐test (with *p* < 0.10 indicating significant heterogeneity), and the effect of covariates were assessed via the QM Wald‐type test. Forest plots were used to visualise the results of the multilevel meta‐analysis, and bubble plots presented the meta‐regression findings. All analyses were conducted using the R packages meta and metafor, ensuring a rigorous statistical framework. A qualified statistician (RH) calculated all analyses to ensure methodological robustness and accuracy.

## RESULTS

### Search results

**Figure 1:**
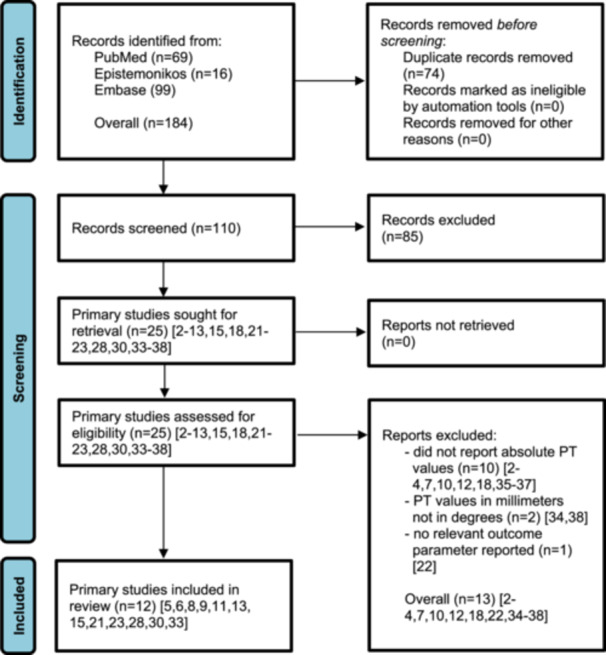
Flow chart diagram. PT, pelvic tilt.

The systematic literature search identified 25 studies [2, 3, 4, 5, 6, 7, 8, 9, 10, 11, 12, 13, 15, 18, 21, 22, 23, 28, 30, 33, 34, 35, 36, 37, 38], which underwent full‐text screening (*κ* = 0.96). Of these, 13 studies [2, 3, 4, 7, 10, 12, 18, 22, 34, 35, 36, 37] were excluded for the following reasons (*κ* = 1.00): (1) 10 studies did not report absolute PT values; [2, 3, 4, 7, 10, 12, 18, 35, 36, 37] (2) two studies provided PT values in millimetres rather than degrees; [34, 38] and (3) one study did not assess the relevant outcome [22]. Consequently, 12 primary studies [5, 6, 8, 9, 11, 13, 15, 21, 23, 28, 30, 33] were included in the multilevel meta‐analysis (Figure 1).

### Characteristics of the included primary studies

The 12 included primary studies [5, 6, 8, 9, 11, 13, 15, 21, 23, 28, 30, 33], published between 2018 and 2025, comprised a total of 680 patients and 785 operated hips. Three of these studies reported on bilateral PAOs [5, 6, 28]. The mean patient age was 29.6 years (range: 16–50 years), with 124 male patients (18.2%). The mean body mass index (BMI) was 23.4 kg/m², ranging from 20.9 to 25.2 kg/m² (Table 1).

**Table 1:** Study and patient characteristics.

Primary study	Journal	Study design	LoE	Indication	Operation	Patients, *N*	Hips, *N*	Age, years ± SD (range)	Male sex, *N* (%)	BMI, kg/m² ± SD (range)	Follow‐up time, months ± SD (range)	Radiological imaging	Method of PT measurement
Cirrincione et al. [5]	*Clinical Orthopaedics and Related Research*	Retrospective cohort study	3	DDH	Bernese PAO	35	35	27.0 ± 8.0	1 (2.8)	NR	15.0 ± 8.0 (11.0–65.0)	AP and lateral radiograph	Manual
					Bernese PAO	20	40						
Curley et al. [6]	*Journal of Pediatric Orthopaedics*	Retrospective cohort study	3	DDH	Bernese PAO	53	53	18.5 ± 4.1	5 (9.4)	NR	12.0	AP radiograph	Manual
					Bernese PAO	21	42	21.3 ± 5.1	1 (2.4)		12.0		
Fischer et al. [8]	*Knee Surgery, Sports Traumatology, Arthroscopy*	Prospective cohort study	3	DDH,BDDH, RT	Modified Bernese PAO	60	60	32.4 ± 7.9	27 (23.9)	23.7 ± 2.5	NR	Lateral radiograph	Manual
					Modified Bernese PAO	45	46	28.7 ± 7.8		23.0 ± 3.1			
					Modified Bernese PAO	8	8	31.4 ± 5.7		25.2 ± 2.5			
Fischer et al. [9]	*Journal of Experimental Orthopaedics*	Prospective cohort study	4	DDH,BDDH, RT	Modified Bernese PAO	34	34	29.0	34 (100)	NR	NR	Lateral radiograph	Manual
					Modified Bernese PAO	34	34	29.0	0 (0)				
Fukushima et al. [11]	*Archives of Orthopaedic and Trauma Surgery*	Prospective cohort study	3	DDH	Bernese PAO	30	30	40.2 ± 1.4	0 (0)	20.9 ± 0.37	NR	Lateral radiograph	Manual
					HAS	30	30	43.1 ± 2.3	0 (0)	22.1 ± 0.54			
Grammatopoulos et al. [13]	*The American Journal of Sports Medicine*	Retrospective case series	4	RT	Anteverting Bernese PAO	42	48	30.0 ± 7.0	2 (4.8)	NR	26.0 ± 17.0	AP radiograph, CT	Manual
Heimann et al. [15]	*Journal of Hip Preservation Surgery*	Prospective cohort study	3	DDH	Bernese PAO	64	71	29.0 ± 9.0	19 (3.0)	NR	216.0 ± 96.0	AP radiograph	Statistical shape modelling
Laboudie et al. [21]	*The American Journal of Sports Medicine*	Retrospective cohort study	2	DDH	Bernese PAO	18	18		NR	NR		CT	CT segmentation
Lerch et al. [23]	*Bone and Joint Open*	Retrospective cohort study	3	DDH	Modified Bernese PAO	29	47	29.0 ± 9.0 (16.0–50.0)	2 (6.9)	25.0 ± 5.0 (18.0–35.0)	5.0 ± 2.0	AP and lateral radiograph, CT	Statistical shape modelling
				RT		33	41	28.0 ± 8.0 (17.0–44.0)	5 (15.2)	24.0 ± 4.0 (16.0–33.0)			
				FAI		24	32	28.0 ± 9.0 (18.0–46.0)	15 (62.5)	24.0 ± 4.0 (19.0–34.0)			
Roussot et al. [28]	*Journal of Hip Preservation Surgery*	Retrospective comparative study	2	DDH	Bernese PAO	32	32	29.0 ± 6.0	4 (12.5)	23.3 ± 3.5	30.0 ± 12.0	AP radiograph	PT was calculated from sacro‐femoral‐pubic‐angle
					Bernese PAO	16	32	27.0 ± 6.0	4 (25.0)	23.2 ± 3.0	31.2 ± 12.0		
Schwarz et al. [30]	*Archives of Orthopaedic and Trauma Surgery*	Experimental case series	4	DDH	Bernese PAO with navigation assistance	27	27	27.6	5 (18.2)	22.8	17.5 (12.0–36.0)	AP radiograph	manual
Tani et al. [33]	*Journal of Orthopaedic Research*	Retrospective cohort study	3	DDH	Rotational PAO	25	25	34.0	0 (0)	NR	24.0	Lateral radiograph	Automatic 2D–3D‐registration

Abbreviations: AP, anteroposterior; BDDH, borderline developmental dysplasia of the hip; BMI, body mass index; CT, computed tomography; DDH, developmental dysplasia of the hip; FAI, femoroacetabular impingement; HAS, hip arthroscopy; LoE, level of evidence; NR, not reported; PAO, periacetabular osteotomy; RT, retrotorsion; SD, standard deviation.

### Quality assessment

**Figure 2:**
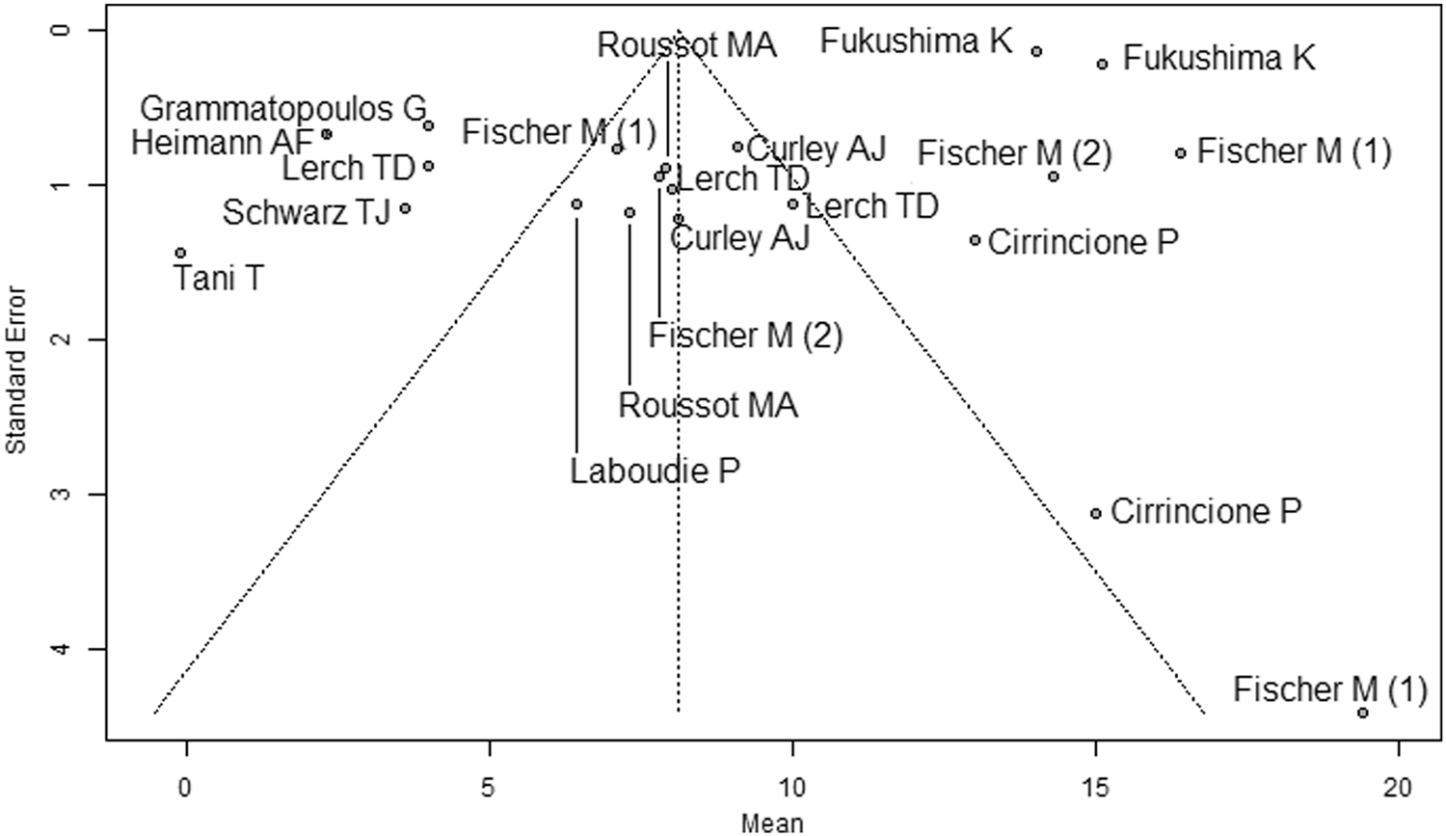
Funnel plot of the mean preoperative pelvic tilt (PT) across 12 studies. The pooled estimate was 8.13° (95% CI: 5.40°–10.86°). Noticeable asymmetry suggests potential publication bias, with a lack of small studies reporting lower effect sizes.

**Figure 3:**
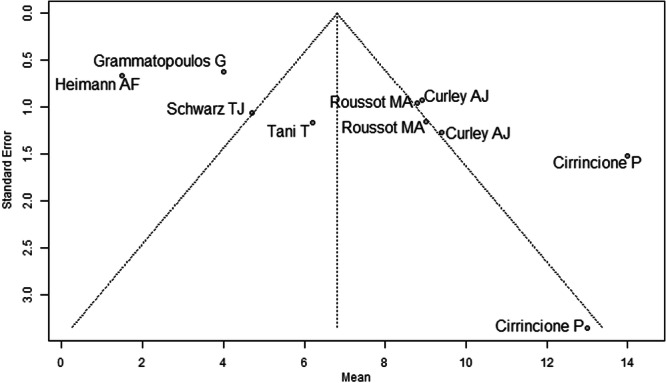
Funnel plot of the mean postoperative pelvic tilt (PT) across eight studies. The pooled estimate was 6.81° (95% CI: 3.38°–10.23°). Mild asymmetry indicates a possible but less pronounced publication bias compared with preoperative values.

**Figure 4:**
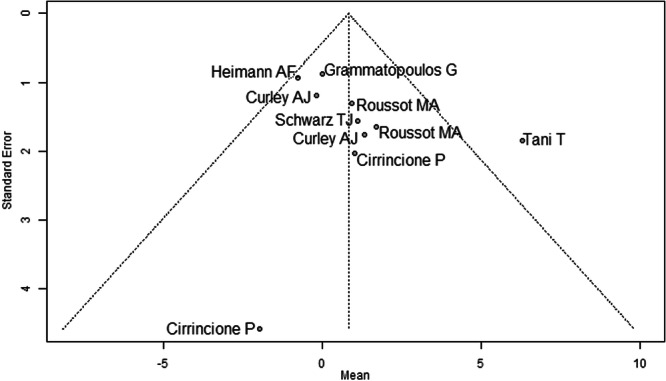
Funnel plot of the mean pelvic tilt (PT) difference before and after surgery across eight studies. The pooled mean change was 0.81° (95% CI: –0.64° to 2.27°), showing no statistically significant alteration; the plot appears largely symmetrical.

Most studies were rated at moderate overall risk of bias using the ROBINS‐I tool [5, 6, 8, 9, 11, 13, 15, 21, 28, 30, 33]. Confounding and selective reporting were the most common concerns, particularly in studies lacking control groups or patient‐reported outcomes. Intervention classification and adherence were consistently well‐reported, with low risk across all studies. Despite some missing data and retrospective designs, no study was judged to be at critical risk of bias (Table 2). Funnel plots were examined for each outcome: preoperative mean pelvic tilt (Figure 2), postoperative mean pelvic tilt (Figure 3), and mean pelvic tilt difference (Figure 4). Figure 2 showed noticeable asymmetry, with a lack of small studies reporting lower effect sizes, suggesting potential publication bias. Figure 3 displayed mild asymmetry, indicating a possible but less pronounced bias. Figure 4 appeared relatively symmetrical, though the presence of a few outliers may still reflect selective reporting. Overall, the findings point to a moderate risk of publication bias across the included studies. Additional funnel plots for mean pelvic tilt values in sitting, standing, and supine positions are provided Supporting Information: Figures S2–S10.

**Table 2:** Risk of bias assessment using the ROBINS‐I tool.

Domain/primary study	Cirrincione et al. [5]	Curley et al. [6]	Fischer et al. [8]	Fischer et al. [9]	Fukushima et al. [11]	Grammatopoulos et al. [13]	Heimann et al. [15]	Laboudie et al. 2022 [21]	Lerch et al. [23]	Roussot et al. [28]	Schwarz et al. [30]	Tani et al. [33]
Bias due to confounding	Moderate	Moderate	Moderate	Moderate	Moderate	Serious	Serious	Moderate	Moderate	Moderate	Serious	Serious
Bias in selection of participants	Low	Low	Low	Low	Low	Moderate	Moderate	Low	Low	Moderate	Low	Low
Bias in classification of interventions	Low	Low	Low	Low	Low	Low	Low	Low	Low	Low	Low	Low
Bias due to deviations from intended interventions	Low	Low	Low	Low	Low	Low	Low	Low	Low	Low	Low	Low
Bias due to missing data	Moderate	Low	Low	Low	Low	Moderate	Moderate	Low	Low	Low	Low	Low
Bias in measurement of outcomes	Low	Low	Low	Low	Low	Low	Low	Low	Low	Low	Low	Low
Bias in selection of the reported result	Moderate	Moderate	Moderate	Moderate	Moderate	Moderate	Moderate	Serious	Low	Moderate	Serious	Serious
Overall Risk of Bias	Moderate	Moderate	Moderate	Moderate	Moderate	Moderate	Moderate	Moderate	Low	Moderate	Moderate	Moderate

*Note*: Possible judgement: low risk, moderate risk, serious risk, and critical risk.

Abbreviation: ROBINS‐I, Risk Of Bias In Non‐randomised Studies of Interventions.

### Single‐arm multilevel meta‐analysis

The overall preoperative PT was 8.13° (95% CI: 5.40°–10.86°). When stratified by position, mean preoperative PT was highest in the sitting position at 18.14° (95% CI: –16.24° to 52.53°), followed by standing at 9.26° (95% CI: 6.24°–12.28°), and lowest in the supine position at 5.10° (95% CI: 0.93°–9.26°).

Postoperatively, the mean overall PT slightly decreased to 6.81° (95% CI: 3.38°–10.23°). In the sitting position, postoperative PT was 14.64° (95% CI: –52.32° to 81.59°); in the standing position, 8.49° (95% CI: 4.96°–12.01°); and in the supine position, 2.76° (95% CI: –13.12° to 18.64°).

**Figure 5:**
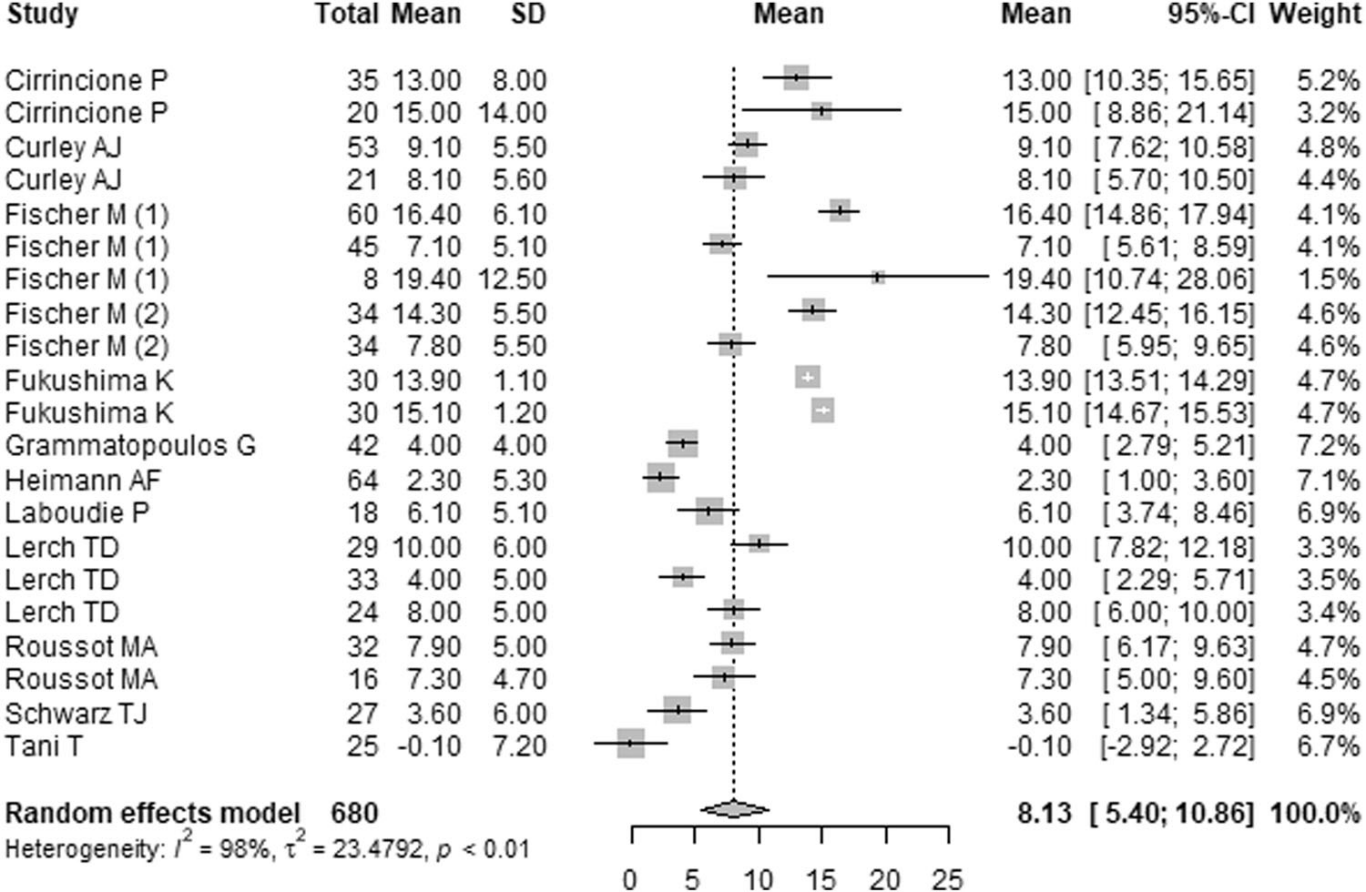
Forest plot of the mean preoperative pelvic tilt (PT) across 12 studies. The pooled mean was 8.13° (95% CI: 5.40°–10.86°), with the highest values in the sitting position (18.14°) and lowest in the supine position (5.10°). Heterogeneity was high (*I*² = 98%, *p* < 0.01). CI, confidence interval; SD, standard deviation.

**Figure 6:**
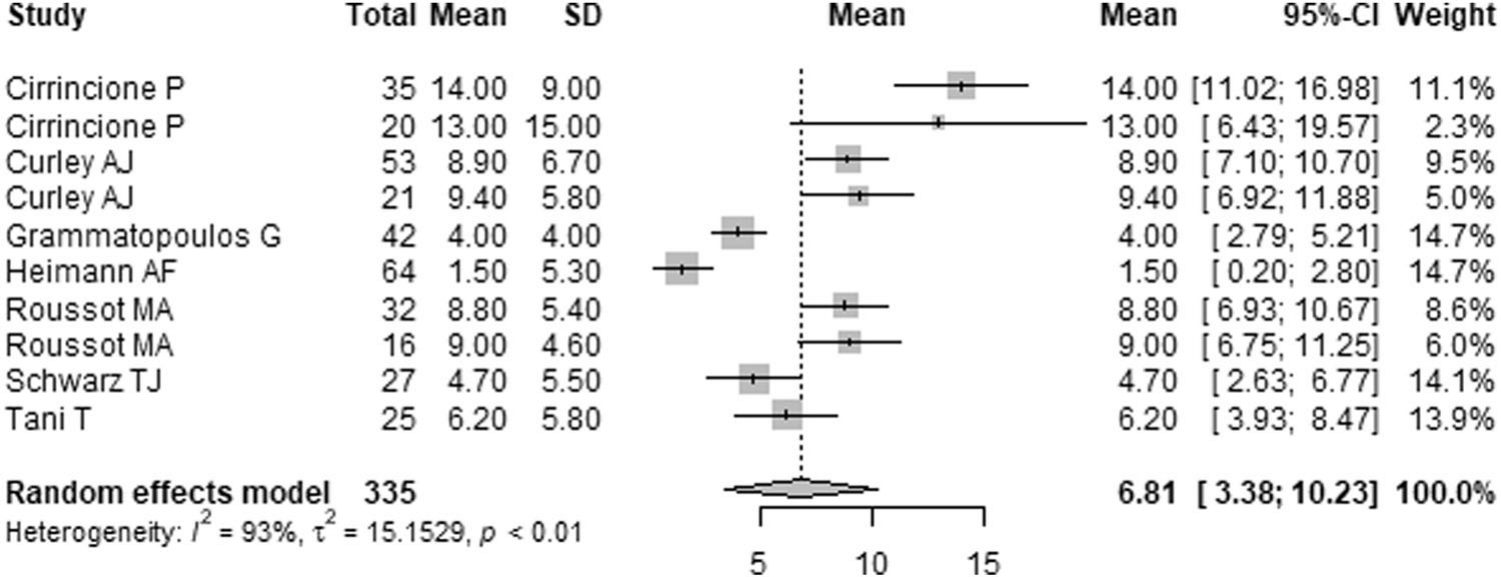
Forest plot of the mean postoperative pelvic tilt (PT) across eight studies. The pooled mean was 6.81° (95% CI: 3.38°–10.23°), with position‐specific means of 14.64° (sitting), 8.49° (standing) and 2.76° (supine). Heterogeneity was high (*I*² = 93%, *p* < 0.01). CI, confidence interval; SD, standard deviation.

**Figure 7:**
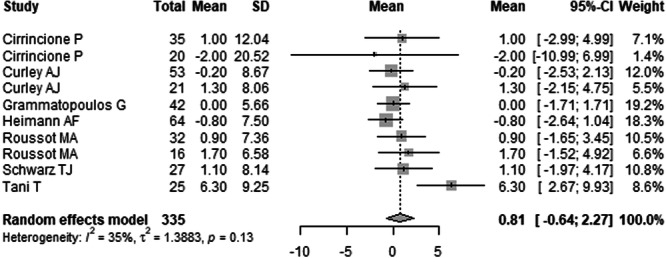
Forest plot of the mean pelvic tilt (PT) difference before and after surgery across eight studies. The pooled mean change was 0.81° (95% CI: –0.64° to 2.27°), indicating a modest and statistically non‐significant decrease in PT. Heterogeneity was low (*I*² = 35%, *p* = 0.13). CI, confidence interval; SD, standard deviation.

The overall mean difference in PT before and after surgery was 0.81° (95% CI: –0.64° to 2.27°), indicating a modest, statistically non‐significant change. Positional analysis showed the greatest change in PT occurred in the sitting position, with a mean difference of 12.01° (95% CI: –45.28° to 69.30°). Standing position changes were minor (mean difference: 1.60°, 95% CI: –0.57° to 3.76°), and PT remained relatively stable in the supine position (mean difference: –0.37°, 95% CI: –5.44° to 4.70°). Further details are presented in Table 3 and Figures 5, 6, 7 (Table 3 and Figures 5, 6, 7). Forest plots illustrating the mean PT values in sitting, standing, and supine position are provided in Supporting Information: Figures S11–S19.

**Table 3:** Results of the single‐arm multilevel meta‐analysis.

Preoperative PT	Mean (in°)	Lower CI	Upper CI
Total	8.13	5.40	10.86
Sitting	18.14	−16.24	52.53
Standing	9.26	6.24	12.28
Supine	5.10	0.93	9.26

Postoperative PT	Mean (in°)	Lower CI	Upper CI
Total	6.81	3.38	10.23
Sitting	14.64	−52.32	81.59
Standing	8.49	4.96	12.01
Supine	2.76	−13.12	18.64

PT difference	Mean (in°)	Lower CI	Upper CI
Total	0.81	−0.64	2.27
Sitting	12.01	−45.28	69.30
Standing	1.60	−0.57	3.76
Supine	−0.37	−5.44	4.70

Abbreviations: CI, confidence interval; PT, pelvic tilt.

### Subgroup analysis

Subgroup analyses revealed no statistically significant differences in PT outcomes based on operative indication (DDH/BDDH vs. other indications) or the number of hips operated on per patient (unilateral vs. bilateral). These findings suggest that neither the underlying diagnosis nor the laterality of surgery had a measurable impact on PT values. Further details are presented in Table 4. Forest plots illustrating the mean PT values from the subgroup analyses are provided in Supporting Information: Figures S20–S36.

**Table 4:** Results of the subgroup analysis.

	Primary studies, *N*	Patients, *N*	Mean preoperative PT		CIs		*τ* ^2^		*I* ^2^		Heterogeneity: *p*		Difference: *p*	
**Preoperative PT**														
Total	21	680	8.13		5.40–10.86		23.48		0.98		<0.0001***			
Subgroup: Surgical laterality														
Unilateral	12	386	9.30		5.41–13.20		28.95		0.97		<0.0001***		0.94	
Bilateral	3	57	9.14		3.73–14.56		28.95		0.62		0.0703			
Subgroup: Operative indication														
DDH/BDDH	13	400	8.14		4.83–11.45		25.15		0.98		<0.0001***			
Other	8	280	8.08		3.59–12.57		25.15		0.97		<0.0001***		0.98	
**Postoperative PT**														
Total	10	335	6.81		3.38–10.23		15.15		0.93		<0.01***			
Subgroup: Surgical laterality														
Unilateral	5	172	8.42		4.77–12.06		9.84		0.86		<0.01***		0.74	
Bilateral	3	57	8.77		4.62–12.92		9.84		0.00		0.53			
Subgroup: Operative indication														
DDH/BDDH	9	293	7.30		3.35–11.25		16.61		0.93		<0.01***			
other	1	42	4.00		−5.50 to 13.50		16.61				‐		0.48	
**PT difference**														
Total	10	335	0.81	−0.64 to 2.27		1.39		0.35		0.13			
Subgroup: surgical laterality													
Unilateral	5	172	1.54	−0.90 to 3.97		2.53		0.56		0.06		0.79	
Bilateral	3	57	1.92	−1.65 to 5.50		2.53		0.00		0.75			
Subgroup: operative indication													
DDH/BDDH	9	293	1.11	−0.84 to 3.05		2.49		0.4		0.10			
Other	1	42	0.00	−4.16 to 4.16		2.49				‐		0.59	

Abbreviations: BDDH, borderline developmental dysplasia of the hip; CI, confidence interval; DDH, developmental dysplasia of the hip; PT, pelvic tilt.

### Meta‐regression analysis

The meta‐regression analysis assessing the predictors patient age, male sex and BMI revealed no statistically significant associations with postoperative PT, except for PT in the sitting position (Table 5). Patient age was identified as a significant predictor of postoperative PT in the sitting position in three included primary studies [5, 6, 33]. The beta coefficient was—4.45, indicating that with each additional year of age, postoperative PT in the sitting position decreased by 4.45°. The 95% confidence interval ranged from –8.48 to –0.41, not crossing zero, which supports the statistical significance of this finding. The p‐value was 0.0454, falling below the conventional threshold of 0.05, further confirming a statistically significant association. Bubble plots illustrating the mean PT values from the meta‐regression analyses for patient age, male sex and BMI are presented in Supporting Information: Figures S37–S63).

**Table 5:** Results of the meta‐regression analysis.

Outcome	Predictor	Primary studies, *N*	Beta coefficient	Lower CI	Upper CI	*p* value
Preoperative PT						
Total	Patient age	20	0.30	−0.25	0.86	0.27
	Male sex	20	0.04	−0.04	0.12	0.27
	BMI	11	2.40	−1.09	5.90	0.15
Sitting	Patient age	8	2.56	−1.34	6.47	0.16
	Male sex	8	0.08	−0.15	0.30	0.44
	BMI	3	6.78	−40.31	53.87	0.32
Standing	Patient age	18	0.28	−0.25	0.82	0.27
	Male sex	18	0.04	−0.04	0.12	0.27
	BMI	11	2.40	−1.09	5.90	0.15
Supine	Patient age	3	1.17	−0.50	2.84	0.07
	Male sex	3	−0.16	−1.58	1.27	0.40
	BMI	0				
Postoperative PT						
Total	Patient age	10	−0.17	−0.83	0.49	0.56
	Male sex	10	−0.08	−0.28	0.13	0.41
	BMI	3	8.73	−26.31	43.77	0.19
Sitting	Patient age	3	−4.45	−8.48	−0.41	0.04*
	Male sex	3	2.52	−90.46	95.50	0.78
	BMI	0				
Standing	Patient age	8	−0.10	−0.76	0.56	0.72
	Male sex	8	−0.04	−0.27	0.20	0.73
	BMI	3	8.73	−26.31	43.77	0.19
Supine	Patient age	2				
	Male sex	2				
	BMI	0				
PT difference						
Total	Patient age	10	0.20	−0.19	0.59	0.27
	Male sex	10	−0.07	−0.20	0.07	0.27
	BMI	3	−0.06	−50.60	50.48	0.99
Sitting	Patient age	3	3.80	−2.53	10.14	0.08
	Male sex	3	−0.85	−92.57	90.86	0.93
	BMI	0				
Standing	Patient age	8	0.28	−0.05	0.62	0.08
	Male sex	8	−0.07	−0.31	0.18	0.53
	BMI	3	−0.06	−50.60	50.48	0.99
Supine	Patient age	2				
	Male sex	2				
	BMI	0				

Abbreviations: BMI, body mass index; CI, confidence interval; PT, pelvic tilt.

## DISCUSSION

### Key findings

This systematic review and single‐arm multilevel meta‐analysis synthesises current evidence on PT changes in patients undergoing PAO. The analysis revealed that overall PT remains relatively stable following PAO, with only minor, non‐significant changes observed across different body positions. Subgroup analyses showed no significant influence of operative indication or surgical laterality on PT outcomes. However, meta‐regression identified patient age as a significant predictor of postoperative PT in the sitting position, with older patients demonstrating lower PT values, which does not seem to be directly caused by PAO.

### Interpretation of key findings

The modest decrease in mean PT following PAO, particularly in the sitting position, suggests that pelvic orientation does not undergo substantial alteration after acetabular realignment. The lack of significant postoperative change supports the hypothesis that PT in this patient population may be governed more by inherent anatomical and spinopelvic characteristics than by surgical modification alone. Notably, studies comparing DDH patients to healthy controls have found standing PT values to be within normal ranges, although a subset of DDH individuals demonstrates increased anterior pelvic tilt, potentially to improve femoral coverage from an unstable hip [17]. Conversely, DDH patients exhibit pronounced posterior pelvic tilt (up to 7°–8°) when transitioning from supine to standing—consistent before and two years after PAO—indicating that such postural change is habitual rather than surgery‐induced [32]. These dynamic tilt variations highlight the importance of assessing PT in functional positions, as static measures may fail to capture biomechanical nuances relevant to joint loading and personalised surgical planning. Of particular note, older age was significantly associated with reduced PT in the sitting position postoperatively. In our meta‐regression analysis, age emerged as a significant predictor of postoperative pelvic tilt in the sitting position, with older patients showing lower tilt values. However, the available data did not allow identification of a single discrete ‘critical age’ at which this change becomes clinically relevant. This suggests that age‐related variation is likely gradual and multifactorial, rather than driven by a sharp threshold. This could reflect age‐related differences in spinal or pelvic flexibility, as well as variations in compensatory mechanisms or functional adaptation after surgery. These physiological changes may affect the pelvis's ability to rotate anteriorly during seated positioning, potentially influencing joint orientation and load distribution even after corrective osteotomy. From a biomechanical perspective, alterations in pelvic tilt following PAO may occur indirectly rather than through direct modification of the pelvic ring. In patients with DDH, anterior pelvic tilt in standing may serve as a compensatory mechanism to enhance anterosuperior coverage, while posterior tilt in sitting can help avoid anterior impingement. By reorienting the acetabular fragment, PAO increases structural coverage and modifies the impingement‐free arc of motion, potentially reducing the need for such compensations. Even though the osteotomy does not disrupt the pelvis as a whole, these changes in local hip joint geometry and soft‐tissue tension may allow subtle adaptations in sagittal pelvic orientation, particularly during activities involving deep hip flexion, such as sitting.

### Discussion of the existing literature

Previous studies have established the biomechanical relevance of PT in total hip arthroplasty, particularly in relation to component positioning and dislocation risk [19]. However, literature on PT changes following PAO remains limited and inconsistent. Some reports suggest mild normalisation of PT postoperatively, while others argue for its general stability, attributing variations primarily to individual factors. Our findings align with recent data indicating that PT is not significantly altered by PAO, but they uniquely contribute evidence regarding age‐related variation in postoperative PT, specifically in the sitting position. Notably, Haertlé et al. [14] demonstrated that PT may be phenotype‐dependent in dysplastic hips, challenging the notion of PT as purely compensatory and supporting its role as an intrinsic anatomical parameter. In parallel, Sakai et al. [29] emphasised the dynamic nature of hip joint behaviour in DDH, showing measurable femoral head translation during weight‐bearing—particularly in borderline cases—which may further explain individual variation in spinopelvic mechanics. Together, these studies support a multifactorial understanding of PT behaviour and reinforce the need to consider patient‐specific morphology and biomechanics when interpreting postoperative alignment. This study builds on earlier meta‐analytic work demonstrating favourable outcomes of PAO in borderline DDH [27] by expanding the focus to biomechanical alignment, offering complementary insights into post‐surgical functional adaptation.

### Clinical implications

Understanding the relationship between PT and patient‐specific factors such as age is important for preoperative planning and postoperative assessment in PAO. The observation that older patients may exhibit lower PT in the sitting position could inform expectations regarding functional posture and spinopelvic interaction. Although PT does not appear to be significantly altered overall by PAO, awareness of patient‐related variability can guide individualised surgical strategy, radiographic evaluation, and rehabilitation planning.

While PAO is primarily designed to increase acetabular coverage in the coronal plane, there is growing recognition that sagittal orientation, including PT, can be indirectly influenced by the reorientation of the acetabular fragment. In selected cases—particularly those with abnormal spinopelvic parameters or dynamic instability—modifying PT may be considered an adjunctive surgical aim. However, PT correction is not a universal objective in PAO and should be approached with caution, as excessive changes may alter spinopelvic balance or compromise functional alignment.

Moreover, since PT influences measurements like the lateral center‐edge angle and acetabular version, accurate interpretation of these parameters pre‐ and postoperatively must consider potential spinopelvic dynamics, especially in older individuals.

### Limitations

Several limitations should be acknowledged. First, the number of studies included in certain analyses—particularly the meta‐regression on age—was small (*n* = 3), limiting statistical power and generalisability. Second, heterogeneity in PT measurement methods and imaging modalities across studies may have introduced variability. Third, most included studies were observational and rated at moderate risk of bias, raising potential concerns about confounding and reporting accuracy.

Additionally, despite stratification by patient position (sitting, standing and supine), variability in how and when PT was measured could have influenced the comparability of results. Lastly, the study focused exclusively on angular PT values and did not assess dynamic or functional spinopelvic motion, which may also be clinically relevant.

The operative technique descriptions in Table 1 were specified in detail, distinguishing between classical Bernese PAO, modified and anteverting Bernese PAO, and rotational PAO. While all procedures aim to improve acetabular coverage and may influence pelvic tilt, subtle biomechanical differences between techniques should be considered when interpreting the pooled results.

### Directions for future research

Future studies should aim to standardise PT measurement protocols and investigate dynamic spinopelvic relationships using motion‐based imaging. Larger, prospective cohorts are needed to validate the influence of age and other patient‐related factors on PT after PAO. Furthermore, incorporating patient‐reported outcomes, gait analysis, and 3D imaging could provide a more comprehensive understanding of how PT contributes to function, satisfaction, and long‐term joint health following PAO.

## CONCLUSION

PT remains largely unchanged after PAO, with no significant differences observed across operative indications or surgical laterality. However, older age was associated with lower postoperative PT in the sitting position, suggesting age‐related biomechanical variation. These findings highlight the need to consider individual patient factors when assessing pelvic alignment in PAO.

## AUTHOR CONTRIBUTIONS

**Nikolai Ramadanov**: Conceptualisation; data curation; formal analysis; investigation; methodology; project administration; resources; software; supervision; validation; visualisation; writing—original draft; writing—review and editing. **Maximilian Voss**: Data curation; formal analysis; investigation. **Maximilian Heinz**: Data curation; formal analysis; investigation. **Robert Hable**: Conceptualisation; methodology; project administration; resources; software; supervision; validation; visualisation; writing—review and editing. **Robert Prill**: Writing—review and editing. **Roland Becker**: Writing—review and editing. **Plamen Penchev**: Writing—review and editing. **Sufian S. Ahmad**: Writing—review and editing.

## CONFLICT OF INTEREST STATEMENT

The authors declare no conflicts of interest.

## ETHICS STATEMENT

The study protocol was registered in the International Prospective Register of Systematic Reviews (PROSPERO) on 12 May 2025 (CRD420251051267).

## Supporting information

Supporting information.

Supporting information.

Supporting information.

Supporting information.

Supporting information.

Supporting information.

Supporting information.

Supporting information.

Supporting information.

Supporting information.

Supporting information.

Supporting information.

Supporting information.

Supporting information.

Supporting information.

Supporting information.

Supporting information.

Supporting information.

Supporting information.

Supporting information.

Supporting information.

Supporting information.

Supporting information.

Supporting information.

Supporting information.

Supporting information.

Supporting information.

Supporting information.

Supporting information.

Supporting information.

Supporting information.

Supporting information.

Supporting information.

Supporting information.

Supporting information.

Supporting information.

Supporting information.

Supporting information.

Supporting information.

Supporting information.

Supporting information.

Supporting information.

Supporting information.

Supporting information.

Supporting information.

Supporting information.

Supporting information.

Supporting information.

Supporting information.

Supporting information.

Supporting information.

Supporting information.

Supporting information.

Supporting information.

Supporting information.

Supporting information.

Supporting information.

Supporting information.

Supporting information.

Supporting information.

Supporting information.

Supporting information.

Supporting information.

Supporting information.

## Data Availability

The data extraction set will be available in the supplementary materials of the published version of this article.
