# Supplementary figures and images for: Pelvic tilt remains unchanged after periacetabular osteotomy: A single‐arm multilevel meta‐analysis and meta‐regression
Source: J Exp Orthop. 2025 Oct 15;12(4):e70453. doi: 10.1002/jeo2.70453 (PMC12527223; doi:10.1002/jeo2.70453)

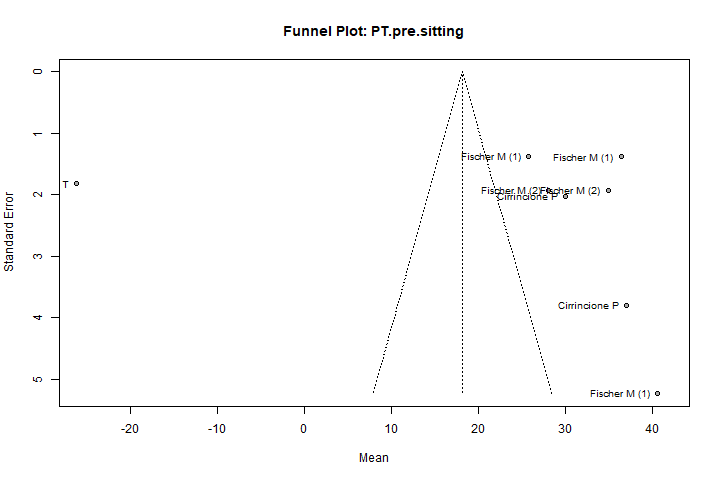

Supplement: Supplementary file 2 — Supporting information. [file JEO2-12-e70453-s043.png]

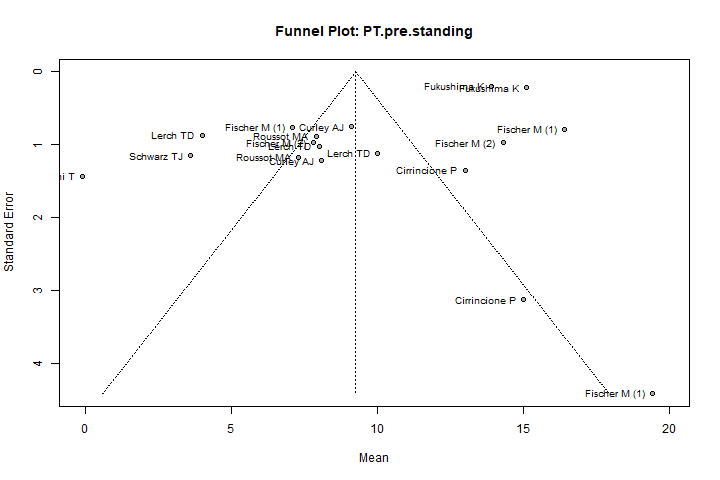

Supplement: Supplementary file 3 — Supporting information. [file JEO2-12-e70453-s021.png]

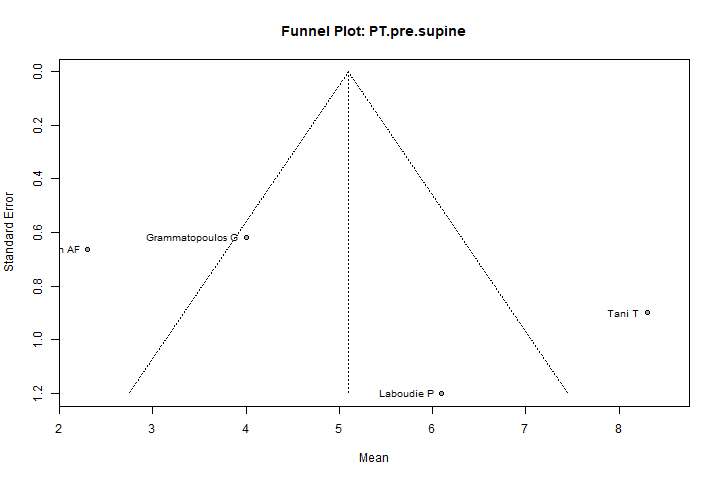

Supplement: Supplementary file 4 — Supporting information. [file JEO2-12-e70453-s044.png]

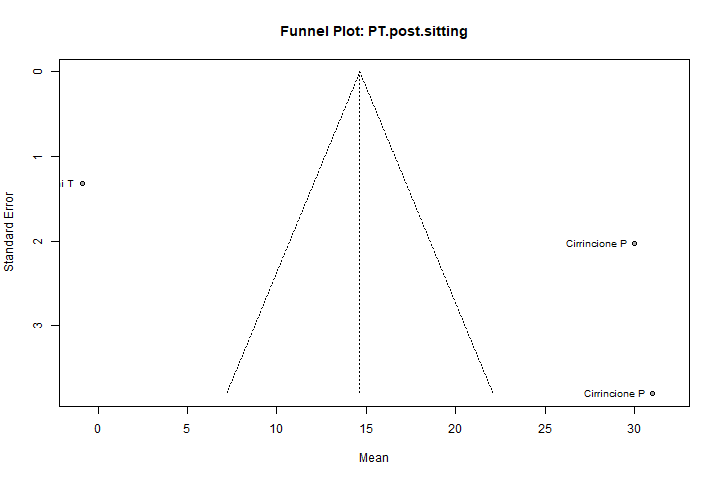

Supplement: Supplementary file 5 — Supporting information. [file JEO2-12-e70453-s008.png]

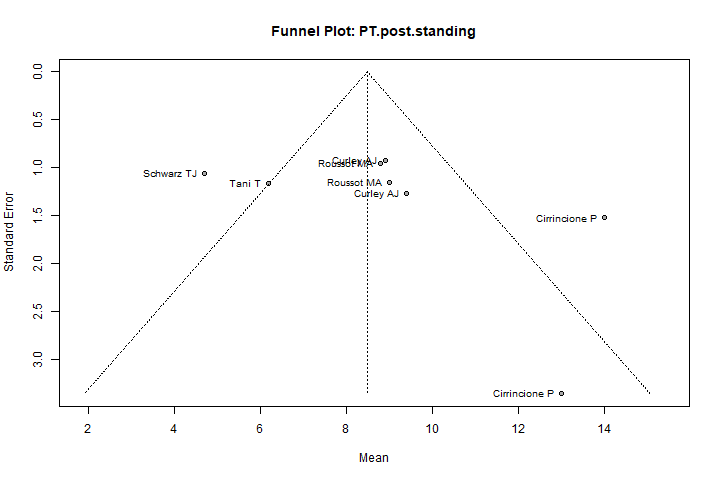

Supplement: Supplementary file 6 — Supporting information. [file JEO2-12-e70453-s060.png]

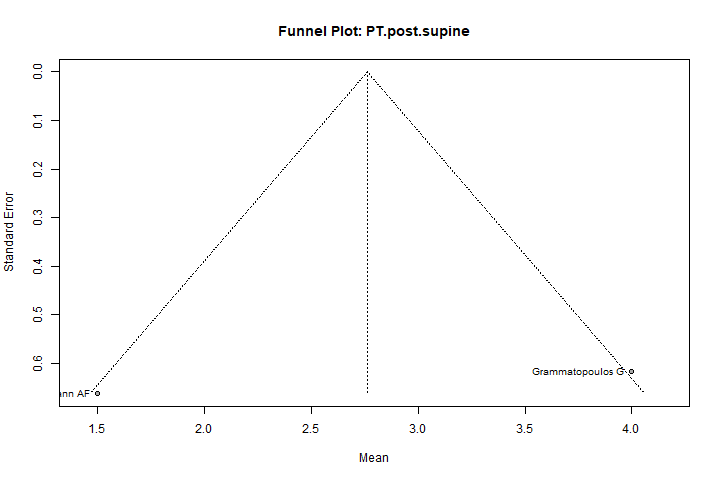

Supplement: Supplementary file 7 — Supporting information. [file JEO2-12-e70453-s040.png]

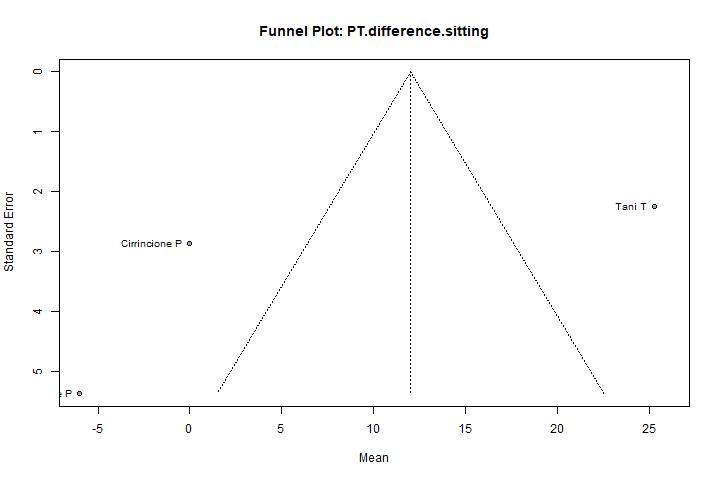

Supplement: Supplementary file 8 — Supporting information. [file JEO2-12-e70453-s057.png]

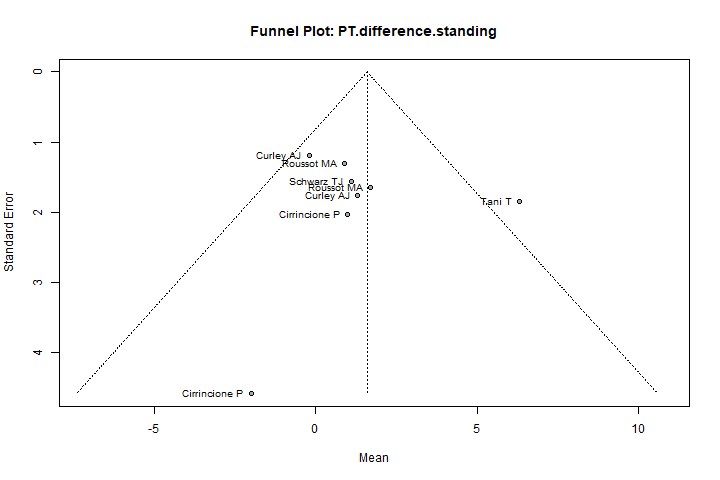

Supplement: Supplementary file 9 — Supporting information. [file JEO2-12-e70453-s023.png]

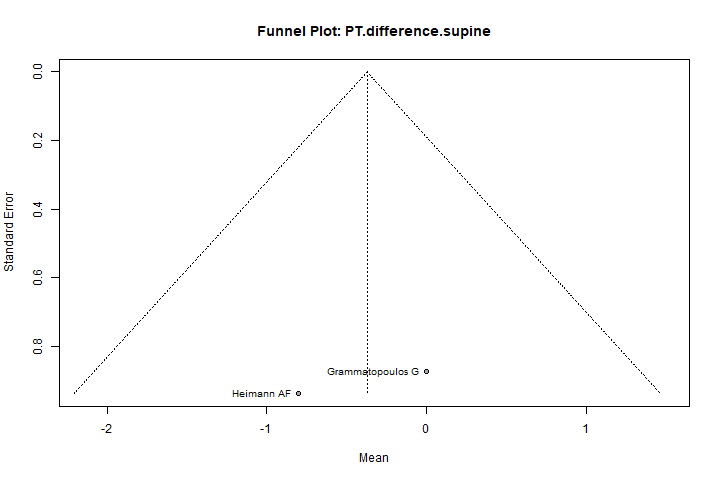

Supplement: Supplementary file 10 — Supporting information. [file JEO2-12-e70453-s012.png]

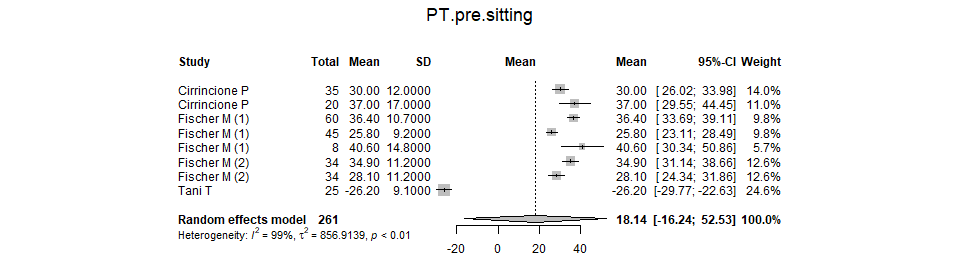

Supplement: Supplementary file 11 — Supporting information. [file JEO2-12-e70453-s052.png]

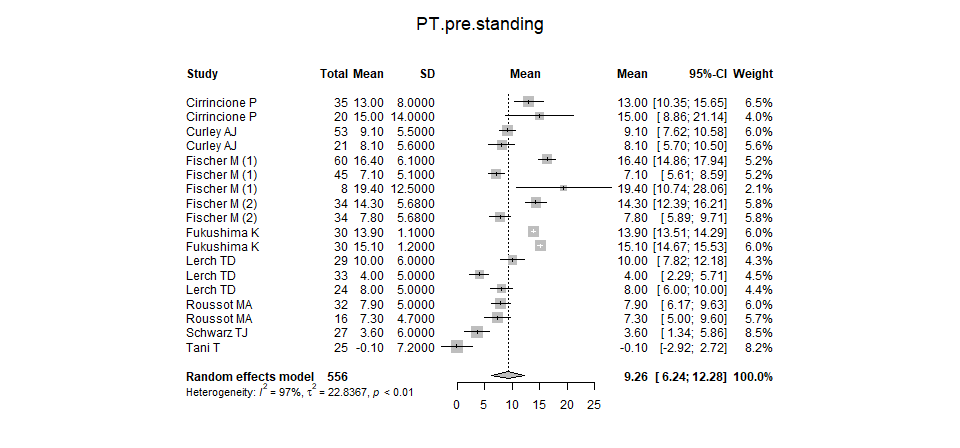

Supplement: Supplementary file 12 — Supporting information. [file JEO2-12-e70453-s059.png]

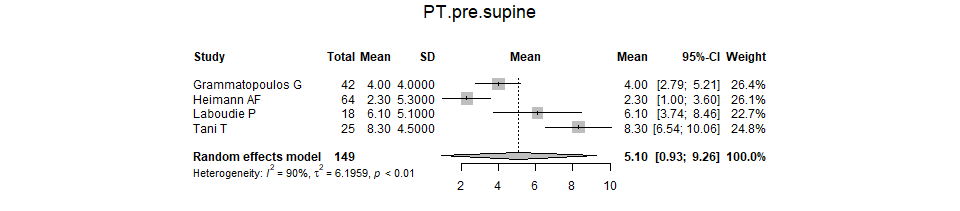

Supplement: Supplementary file 13 — Supporting information. [file JEO2-12-e70453-s013.png]

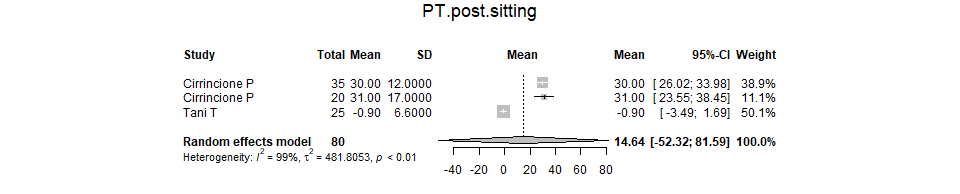

Supplement: Supplementary file 14 — Supporting information. [file JEO2-12-e70453-s004.png]

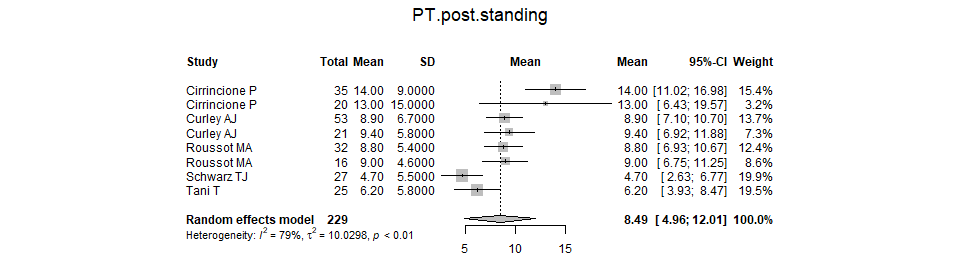

Supplement: Supplementary file 15 — Supporting information. [file JEO2-12-e70453-s017.png]

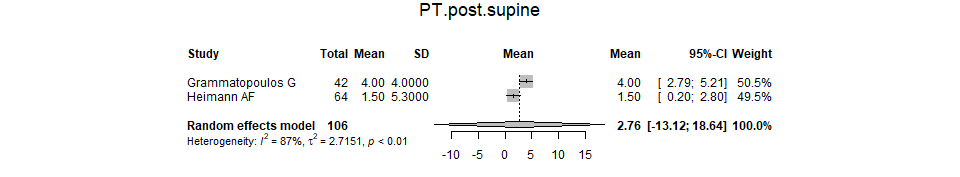

Supplement: Supplementary file 16 — Supporting information. [file JEO2-12-e70453-s063.png]

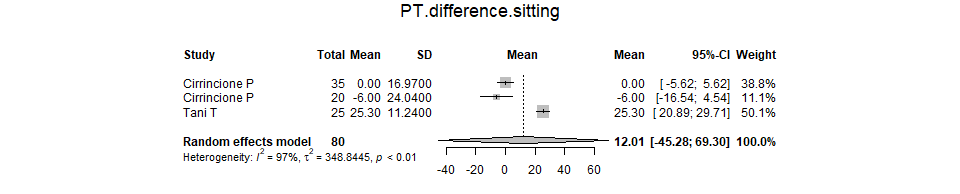

Supplement: Supplementary file 17 — Supporting information. [file JEO2-12-e70453-s011.png]

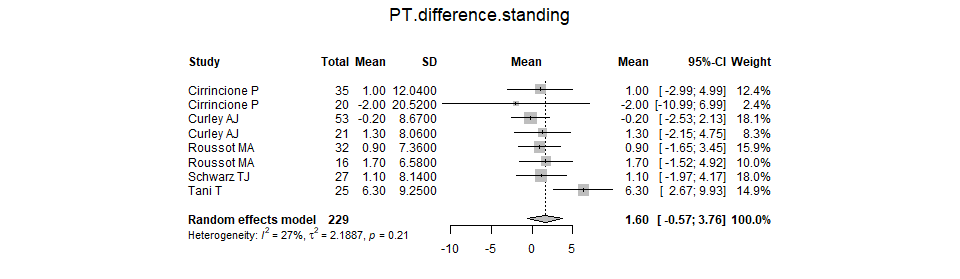

Supplement: Supplementary file 18 — Supporting information. [file JEO2-12-e70453-s010.png]

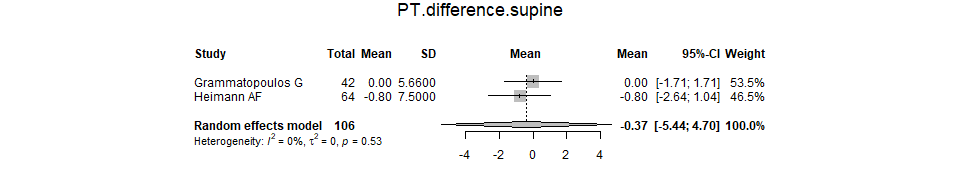

Supplement: Supplementary file 19 — Supporting information. [file JEO2-12-e70453-s025.png]

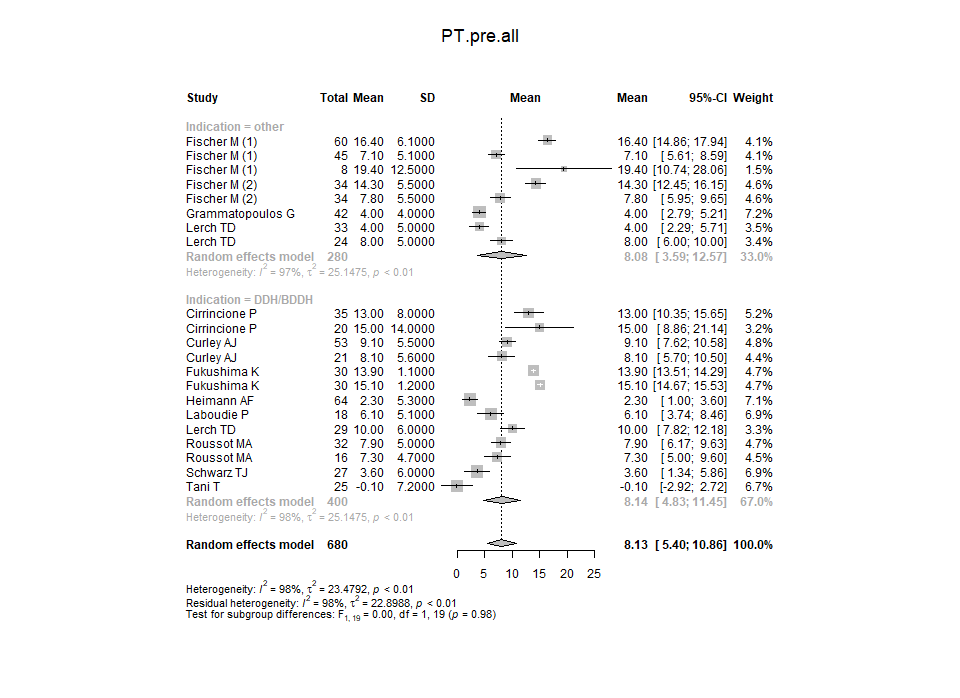

Supplement: Supplementary file 20 — Supporting information. [file JEO2-12-e70453-s009.png]

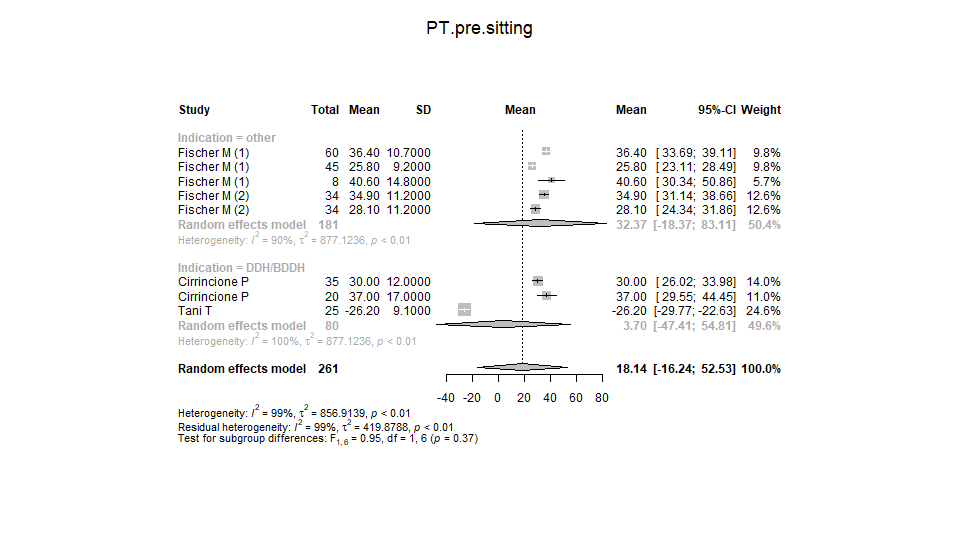

Supplement: Supplementary file 21 — Supporting information. [file JEO2-12-e70453-s041.png]

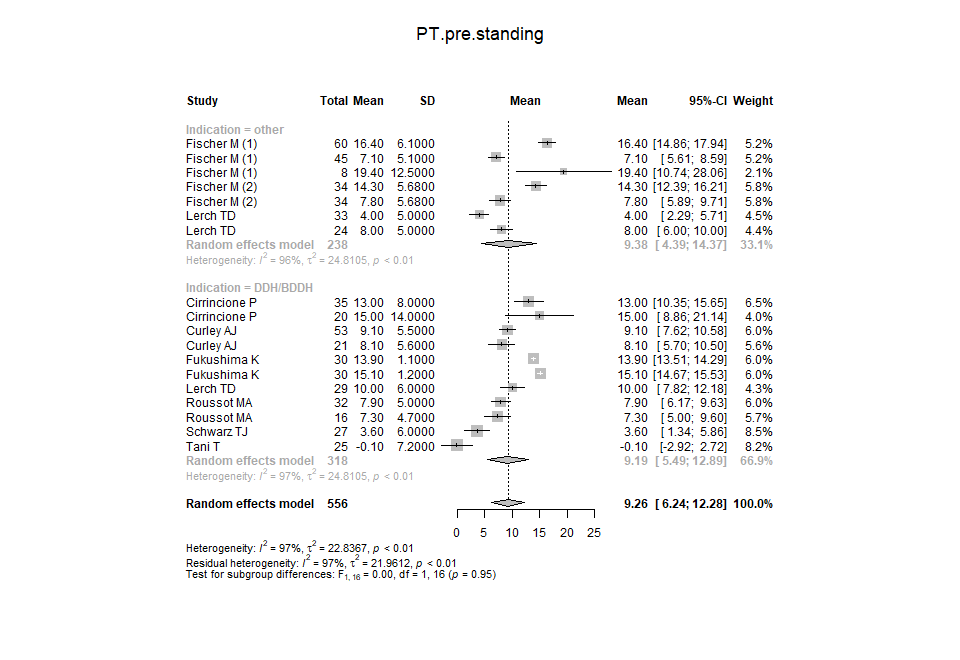

Supplement: Supplementary file 22 — Supporting information. [file JEO2-12-e70453-s033.png]

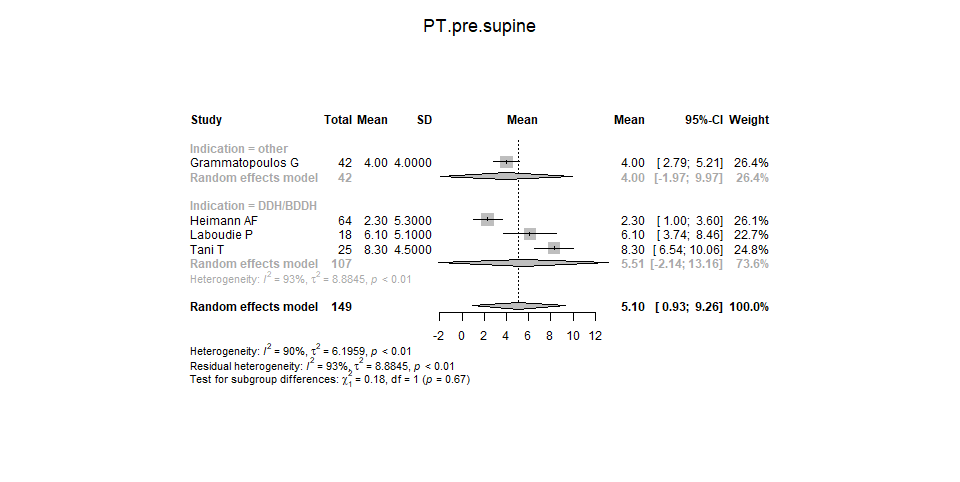

Supplement: Supplementary file 23 — Supporting information. [file JEO2-12-e70453-s054.png]

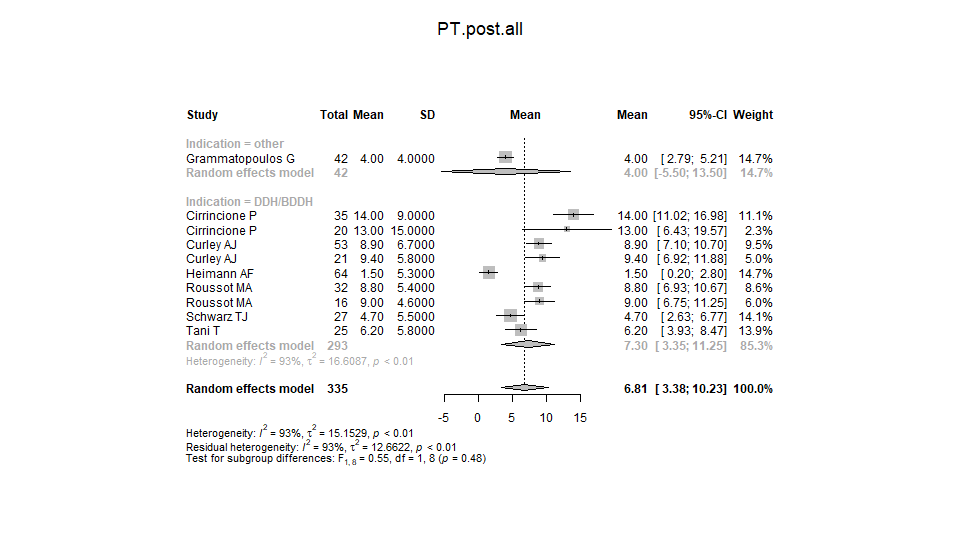

Supplement: Supplementary file 24 — Supporting information. [file JEO2-12-e70453-s056.png]

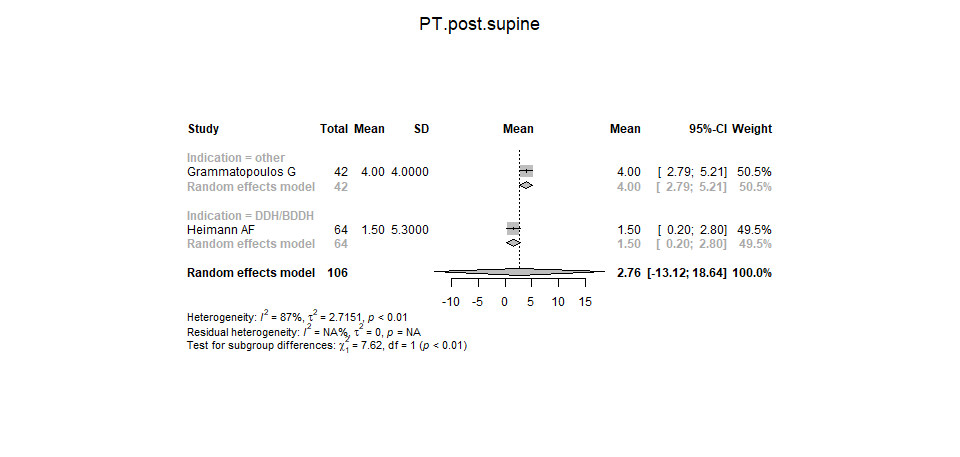

Supplement: Supplementary file 25 — Supporting information. [file JEO2-12-e70453-s015.png]

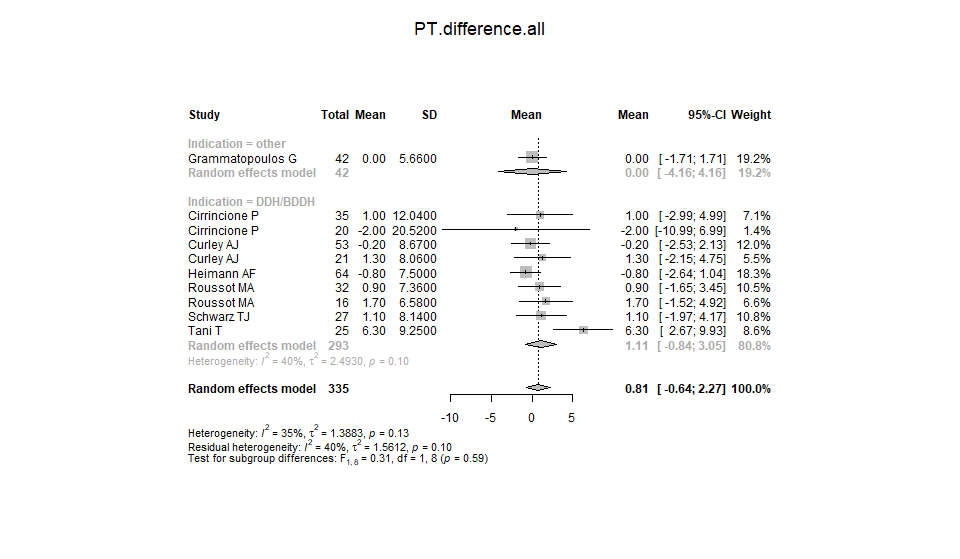

Supplement: Supplementary file 26 — Supporting information. [file JEO2-12-e70453-s042.png]

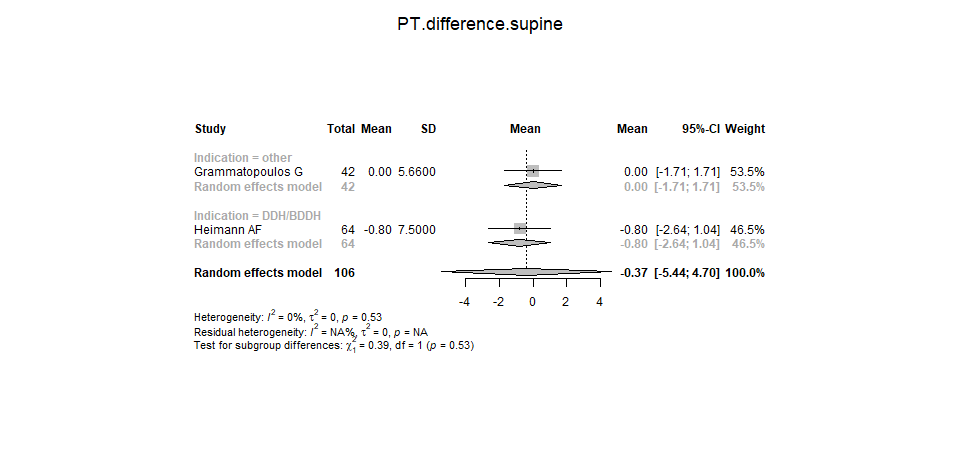

Supplement: Supplementary file 27 — Supporting information. [file JEO2-12-e70453-s022.png]

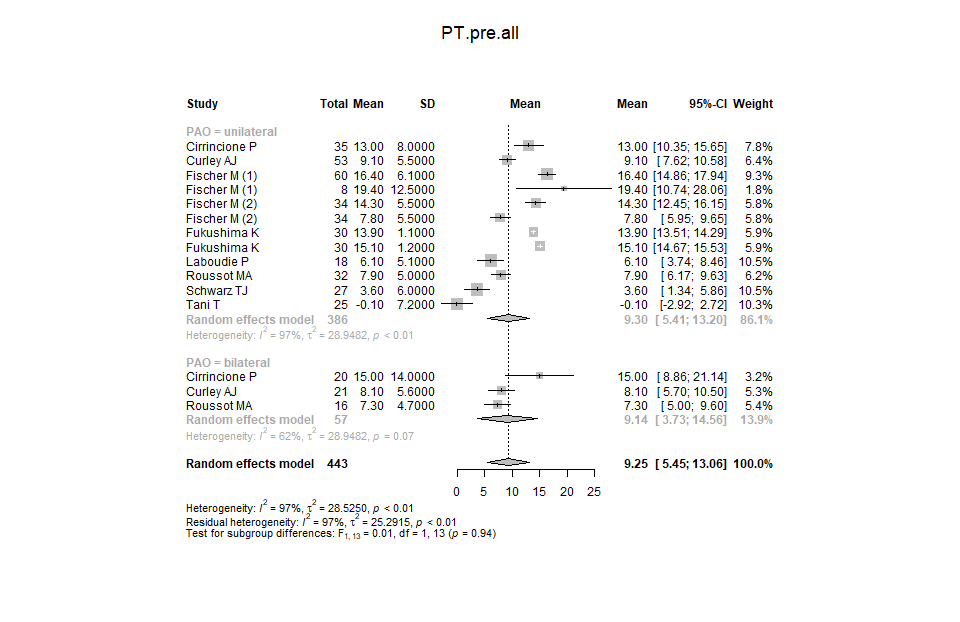

Supplement: Supplementary file 28 — Supporting information. [file JEO2-12-e70453-s027.png]

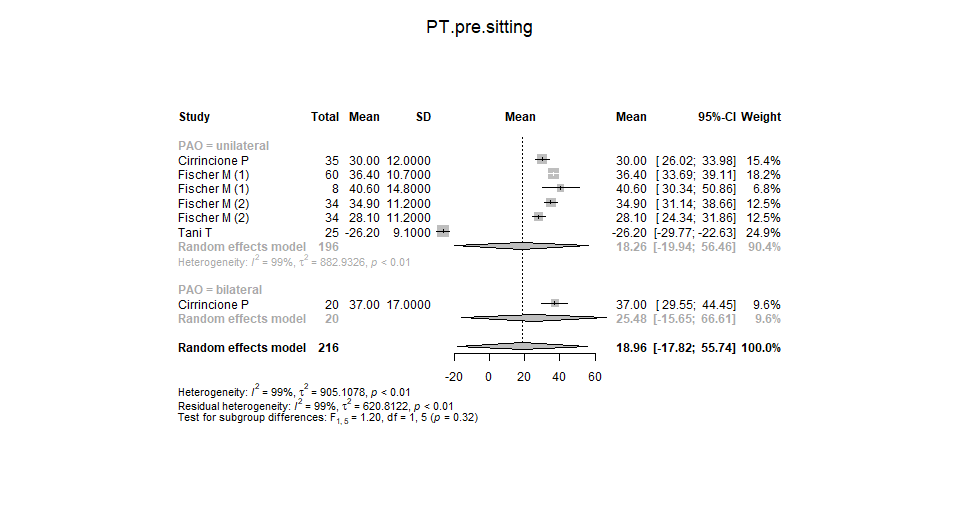

Supplement: Supplementary file 29 — Supporting information. [file JEO2-12-e70453-s036.png]

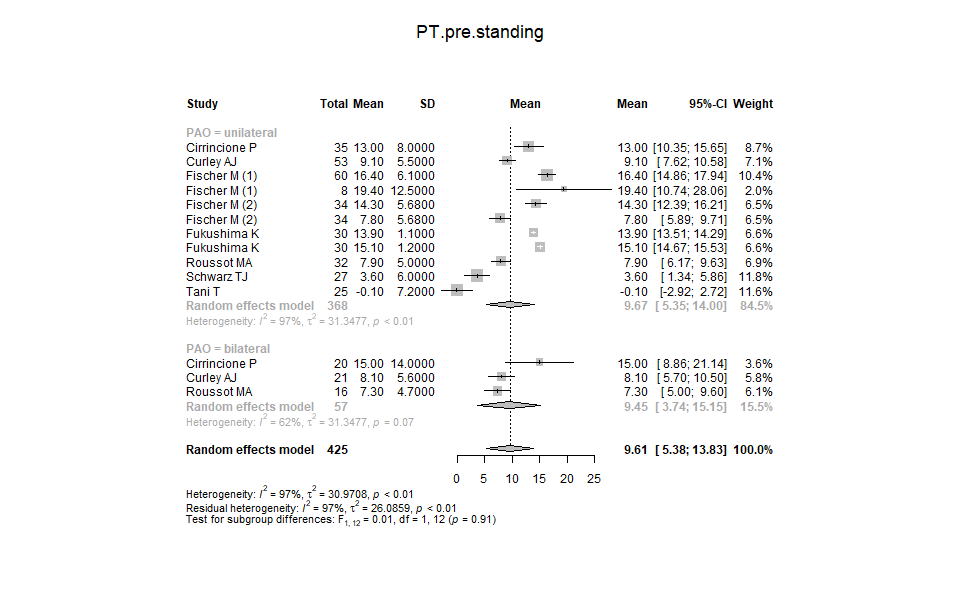

Supplement: Supplementary file 30 — Supporting information. [file JEO2-12-e70453-s007.png]

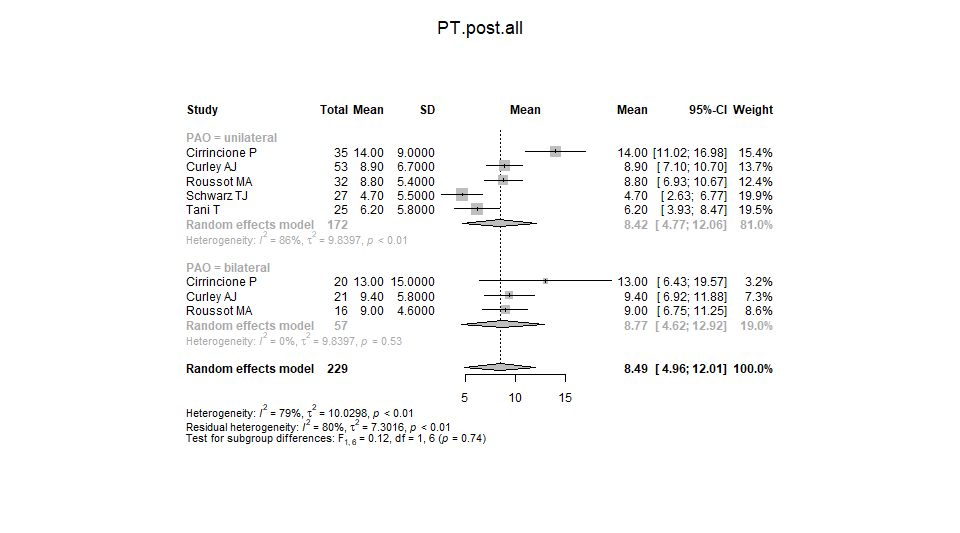

Supplement: Supplementary file 31 — Supporting information. [file JEO2-12-e70453-s030.png]

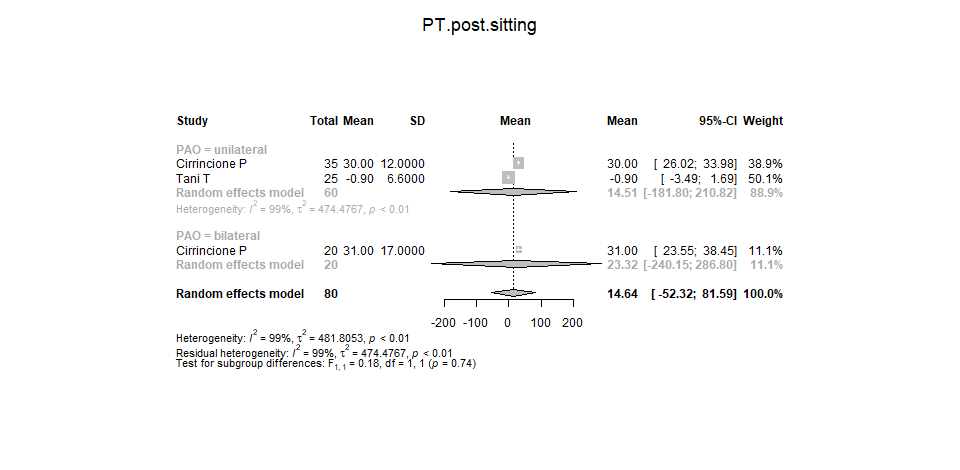

Supplement: Supplementary file 32 — Supporting information. [file JEO2-12-e70453-s003.png]

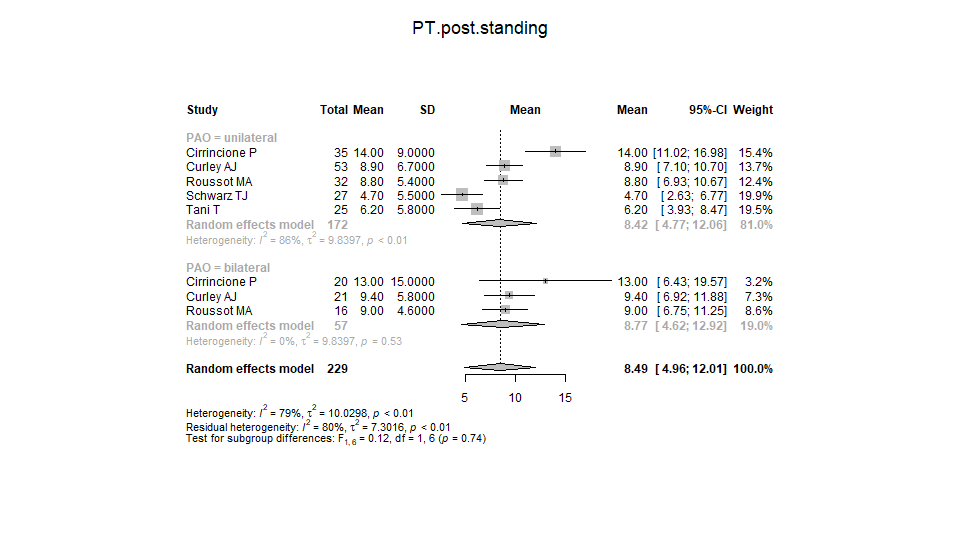

Supplement: Supplementary file 33 — Supporting information. [file JEO2-12-e70453-s047.png]

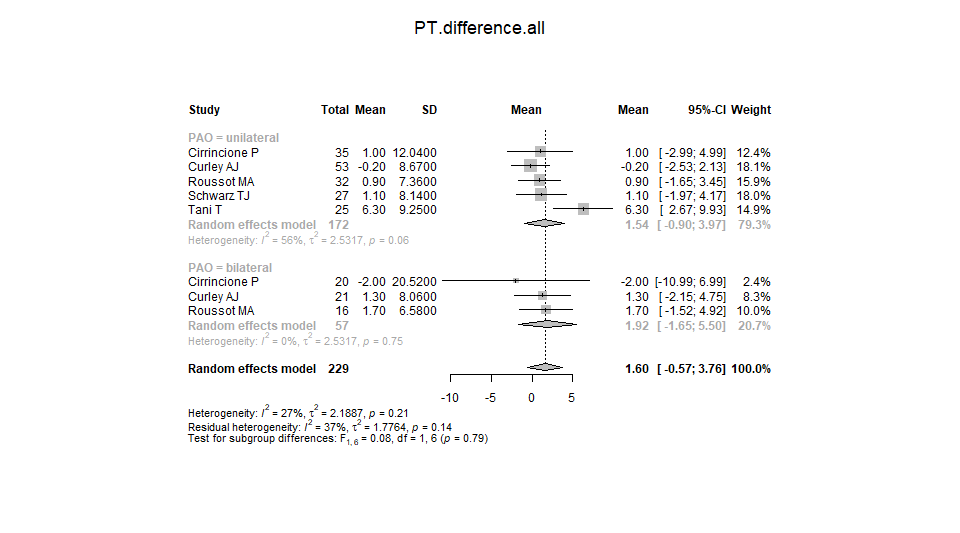

Supplement: Supplementary file 34 — Supporting information. [file JEO2-12-e70453-s016.png]

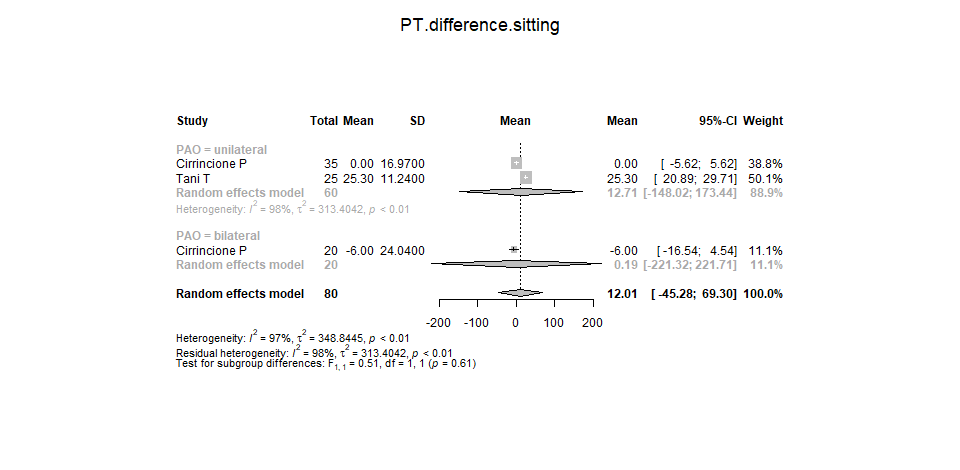

Supplement: Supplementary file 35 — Supporting information. [file JEO2-12-e70453-s029.png]

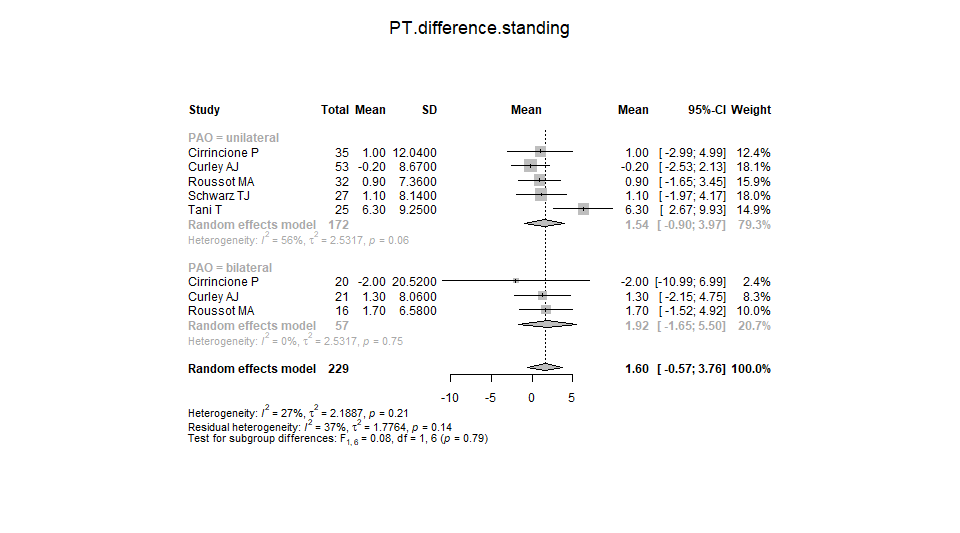

Supplement: Supplementary file 36 — Supporting information. [file JEO2-12-e70453-s019.png]

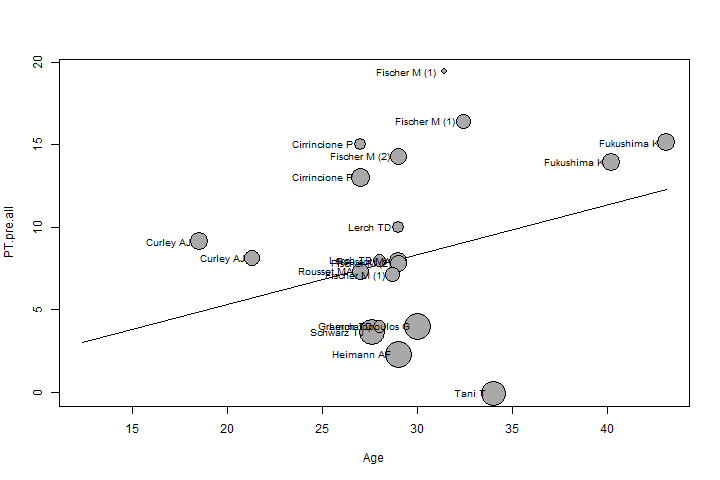

Supplement: Supplementary file 37 — Supporting information. [file JEO2-12-e70453-s020.png]

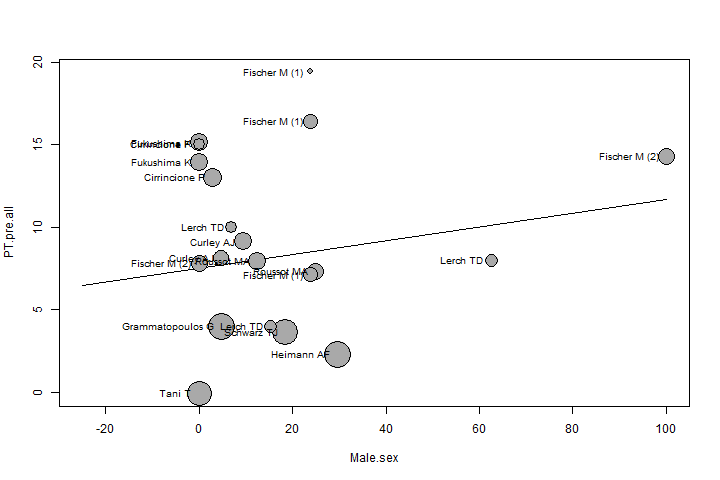

Supplement: Supplementary file 38 — Supporting information. [file JEO2-12-e70453-s061.png]

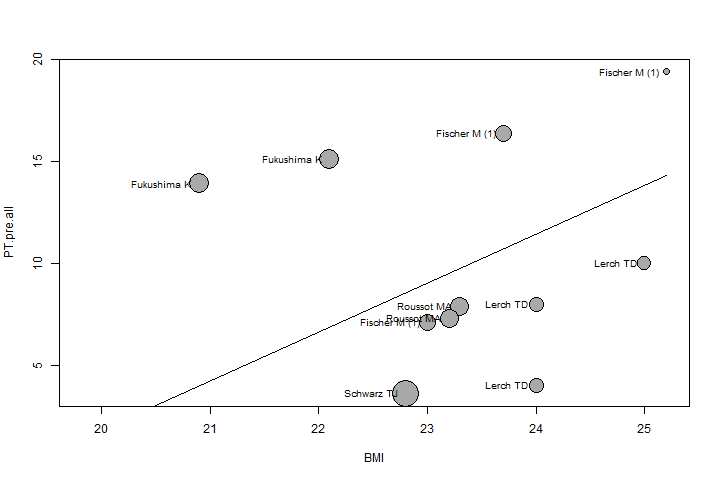

Supplement: Supplementary file 39 — Supporting information. [file JEO2-12-e70453-s032.png]

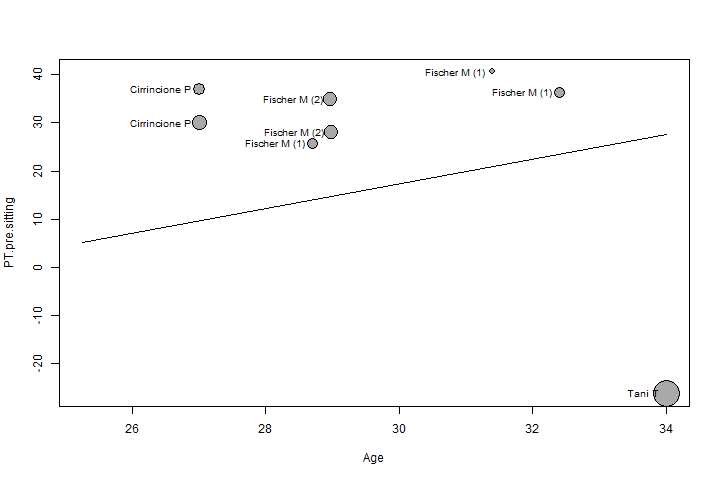

Supplement: Supplementary file 40 — Supporting information. [file JEO2-12-e70453-s053.png]

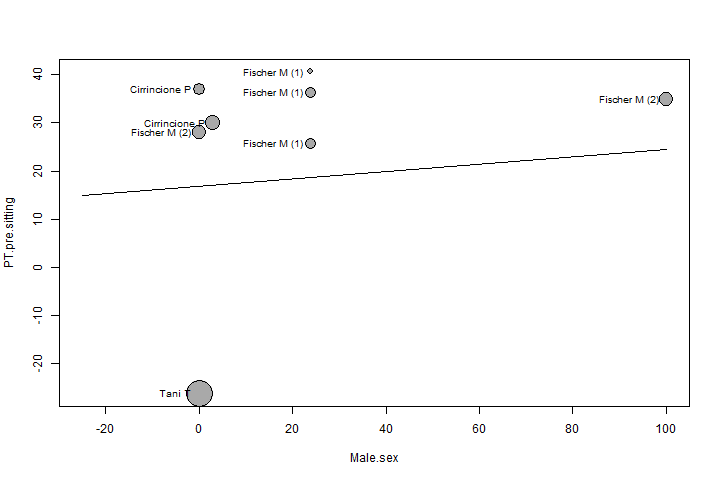

Supplement: Supplementary file 41 — Supporting information. [file JEO2-12-e70453-s018.png]

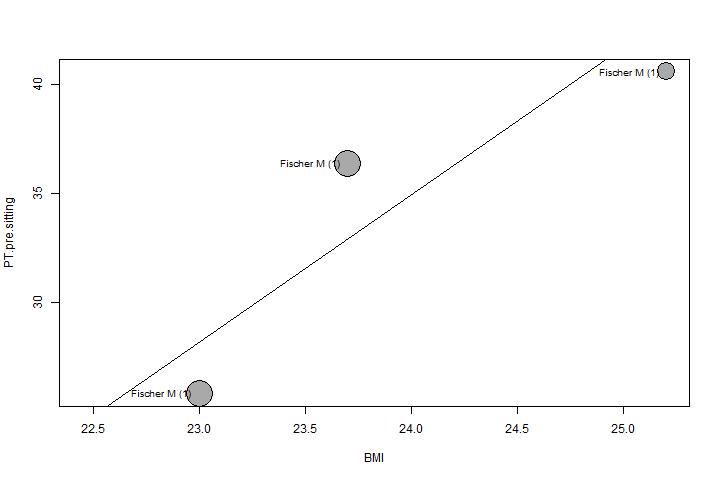

Supplement: Supplementary file 42 — Supporting information. [file JEO2-12-e70453-s001.png]

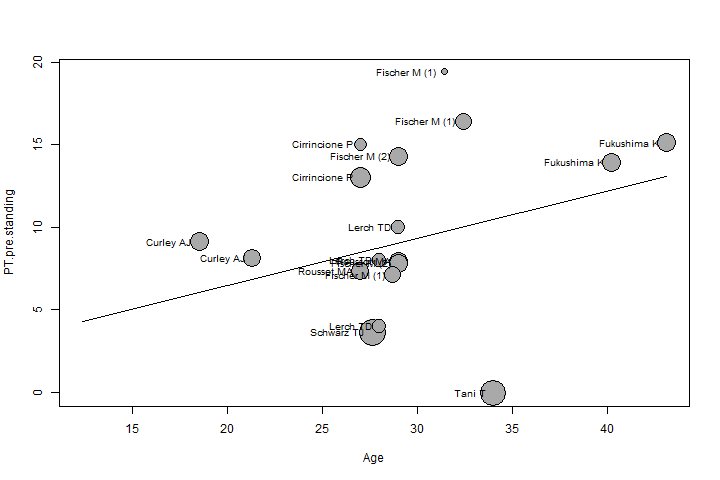

Supplement: Supplementary file 43 — Supporting information. [file JEO2-12-e70453-s002.png]

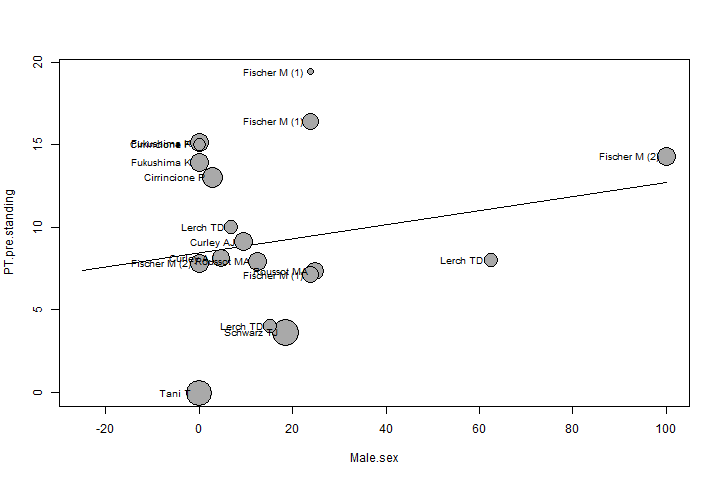

Supplement: Supplementary file 44 — Supporting information. [file JEO2-12-e70453-s048.png]

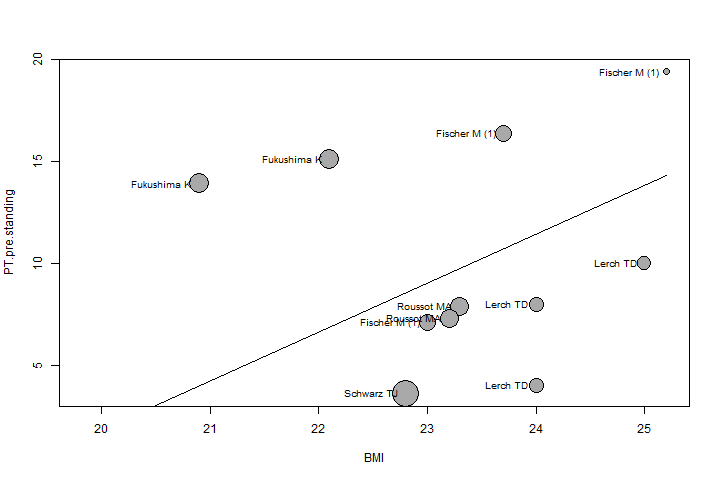

Supplement: Supplementary file 45 — Supporting information. [file JEO2-12-e70453-s024.png]

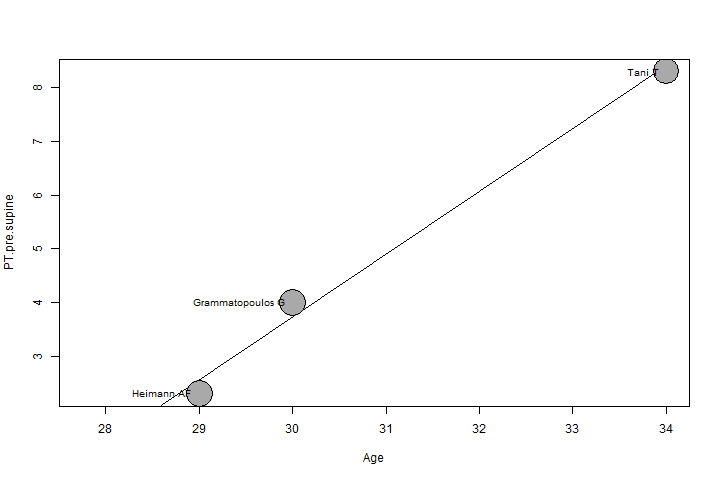

Supplement: Supplementary file 46 — Supporting information. [file JEO2-12-e70453-s031.png]

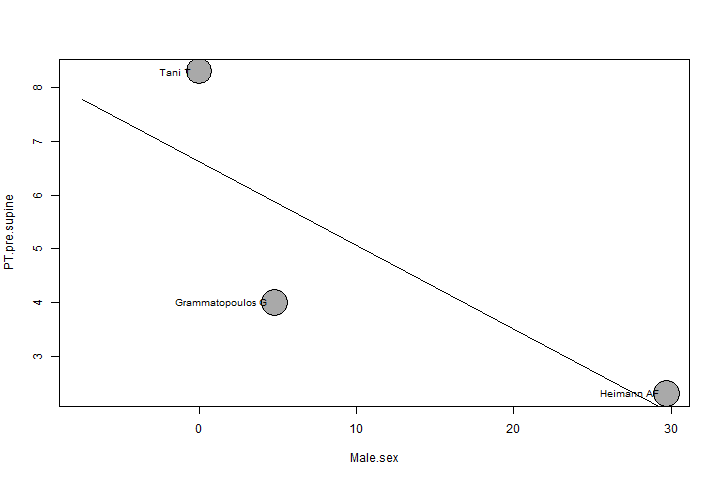

Supplement: Supplementary file 47 — Supporting information. [file JEO2-12-e70453-s039.png]

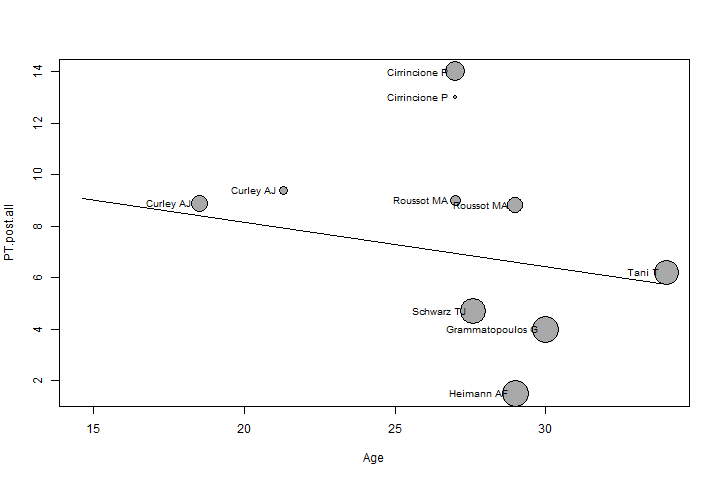

Supplement: Supplementary file 48 — Supporting information. [file JEO2-12-e70453-s034.png]

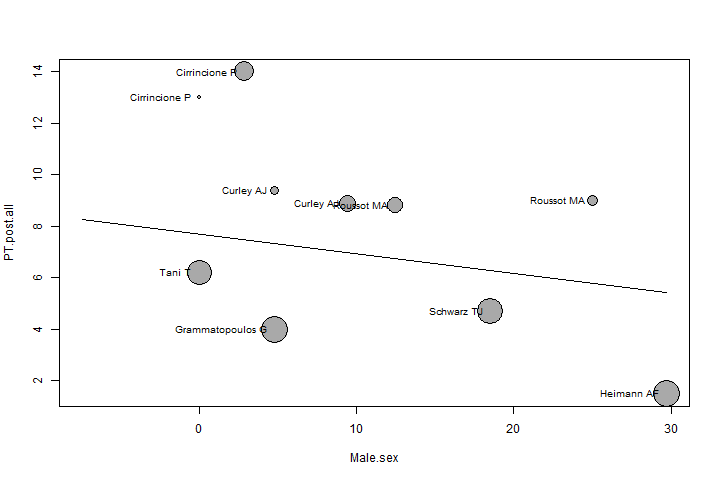

Supplement: Supplementary file 49 — Supporting information. [file JEO2-12-e70453-s064.png]

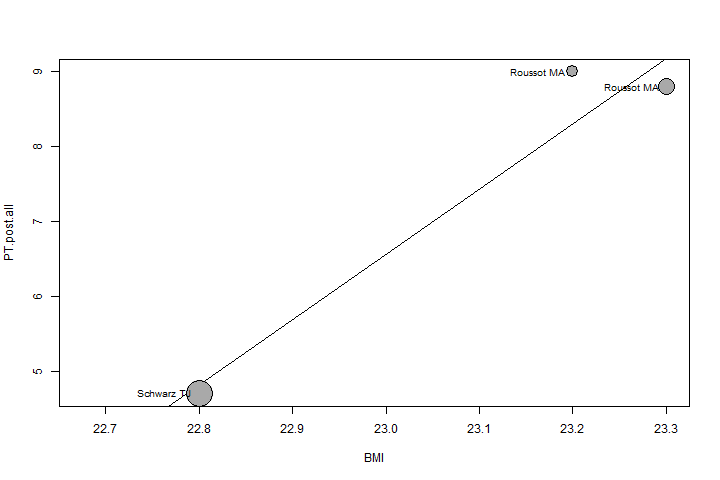

Supplement: Supplementary file 50 — Supporting information. [file JEO2-12-e70453-s028.png]

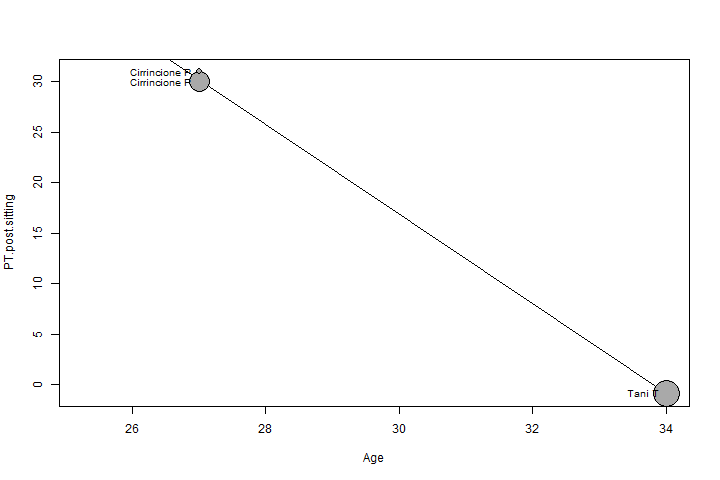

Supplement: Supplementary file 51 — Supporting information. [file JEO2-12-e70453-s045.png]

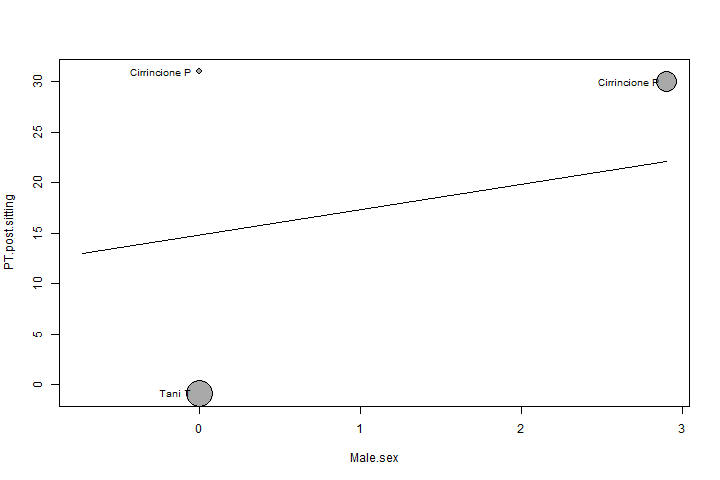

Supplement: Supplementary file 52 — Supporting information. [file JEO2-12-e70453-s037.png]

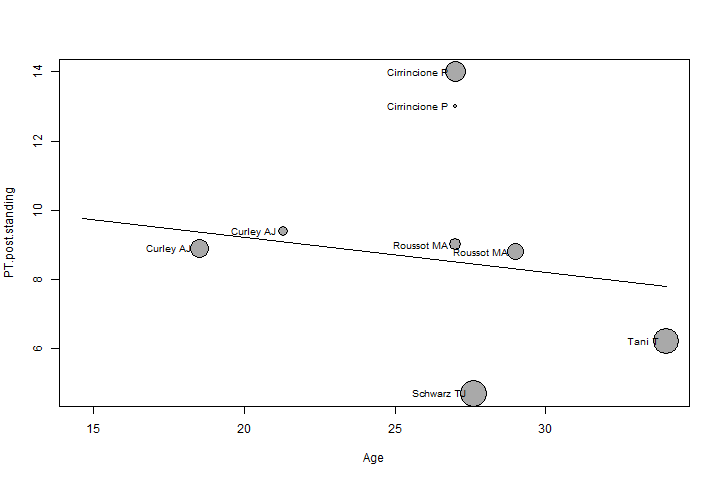

Supplement: Supplementary file 53 — Supporting information. [file JEO2-12-e70453-s049.png]

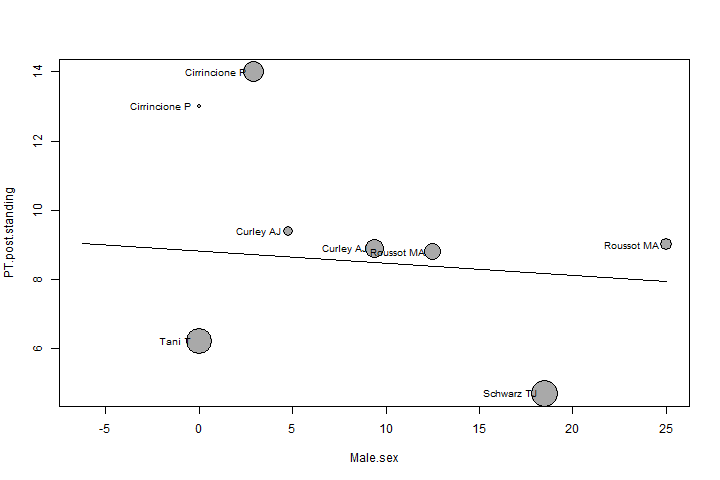

Supplement: Supplementary file 54 — Supporting information. [file JEO2-12-e70453-s014.png]

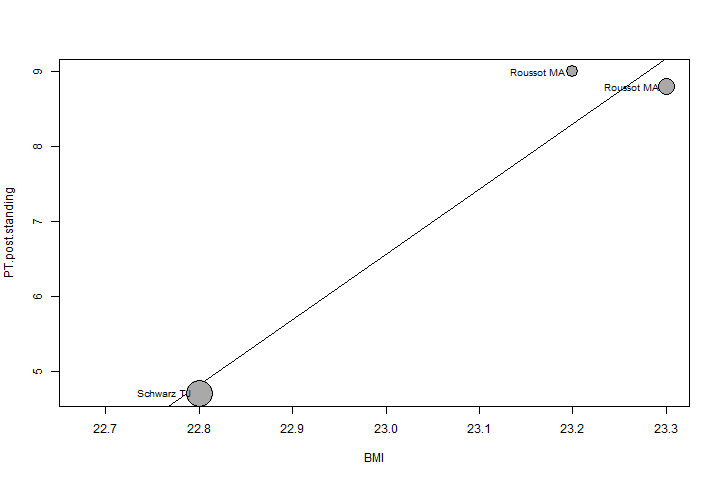

Supplement: Supplementary file 55 — Supporting information. [file JEO2-12-e70453-s005.png]

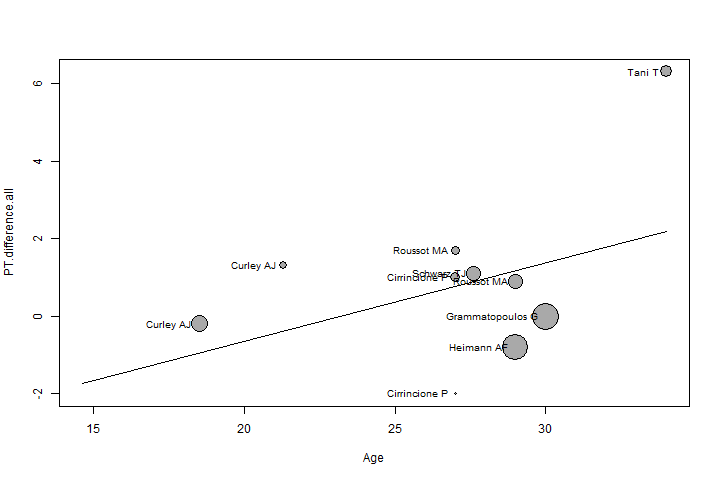

Supplement: Supplementary file 56 — Supporting information. [file JEO2-12-e70453-s006.png]

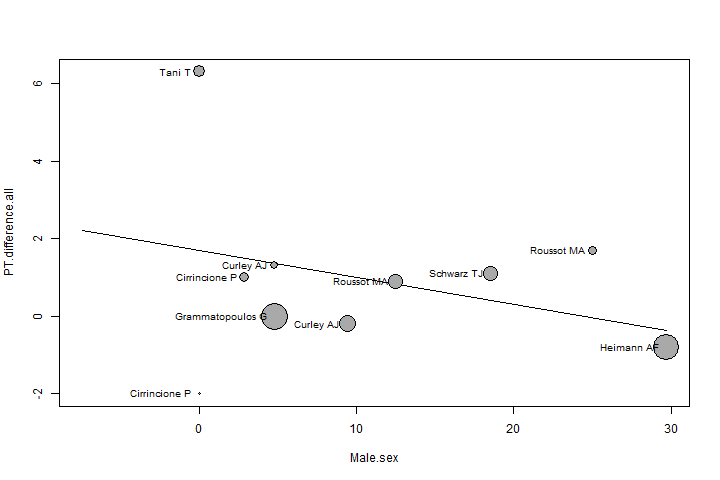

Supplement: Supplementary file 57 — Supporting information. [file JEO2-12-e70453-s051.png]

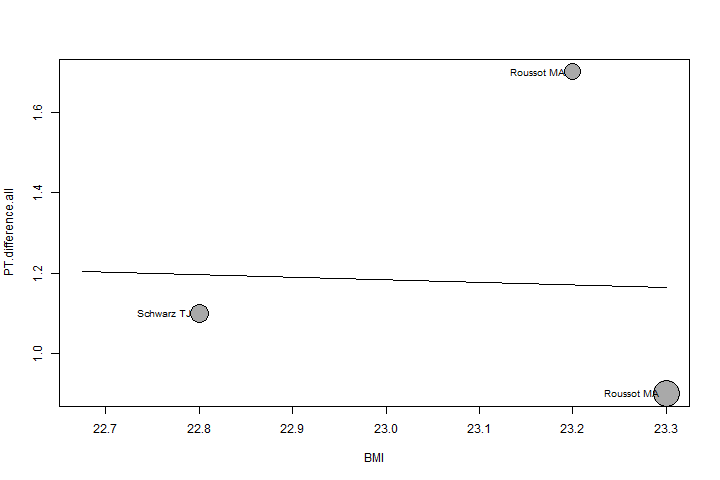

Supplement: Supplementary file 58 — Supporting information. [file JEO2-12-e70453-s050.png]

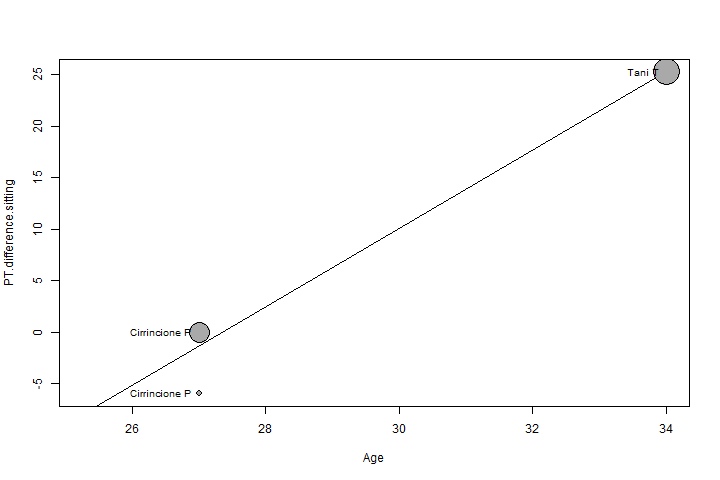

Supplement: Supplementary file 59 — Supporting information. [file JEO2-12-e70453-s062.png]

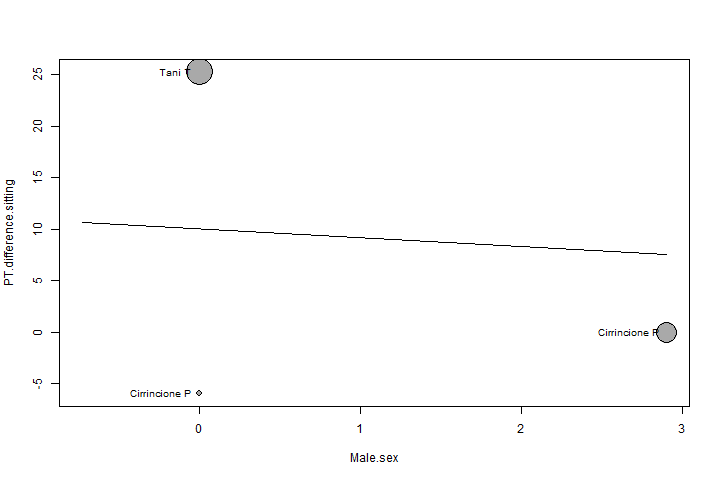

Supplement: Supplementary file 60 — Supporting information. [file JEO2-12-e70453-s055.png]

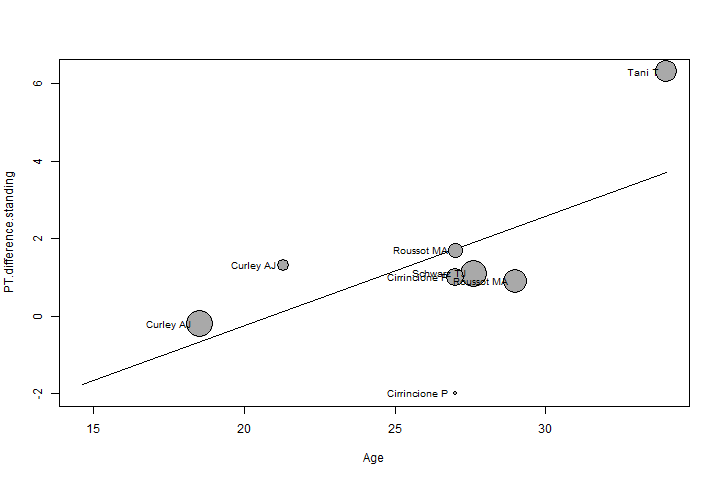

Supplement: Supplementary file 61 — Supporting information. [file JEO2-12-e70453-s035.png]

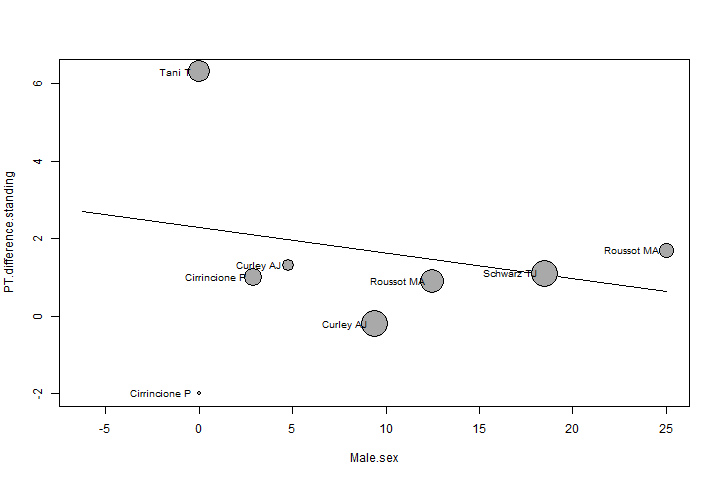

Supplement: Supplementary file 62 — Supporting information. [file JEO2-12-e70453-s046.png]

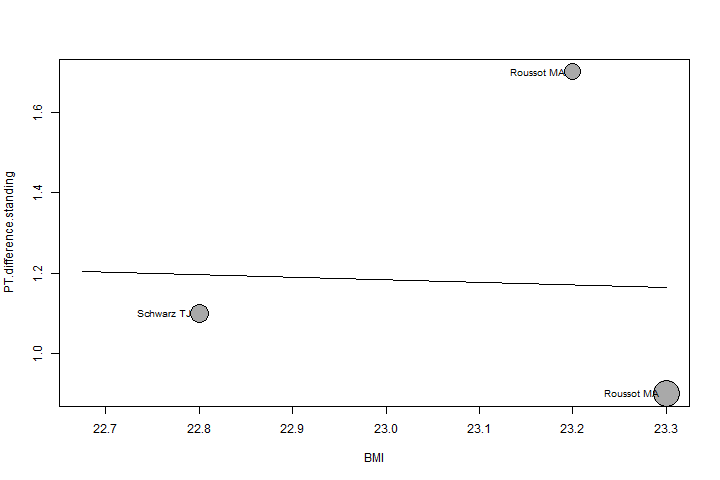

Supplement: Supplementary file 63 — Supporting information. [file JEO2-12-e70453-s058.png]
