# Supplementary material for: Pelvic tilt remains unchanged after periacetabular osteotomy: A single‐arm multilevel meta‐analysis and meta‐regression
Source: J Exp Orthop. 2025 Oct 15;12(4):e70453. doi: 10.1002/jeo2.70453 (PMC12527223; doi:10.1002/jeo2.70453)
Supplement: Supplementary file 64 — Supporting information. [file JEO2-12-e70453-s038.docx]

**SUPPLEMENTARY FIGURES**

**Supplementary Figure S1:** PRISMA Checklist.

**Supplementary Figure S2:** Funnel plot of mean preoperative pelvic tilt (PT) in the sitting position. Mean preoperative PT: 18.14° (95% CI: –16.24° to 52.53°). The funnel plot shows noticeable asymmetry, suggesting possible publication bias.

**Supplementary Figure S3:** Funnel plot of mean preoperative pelvic tilt (PT) in the standing position. Mean preoperative PT: 9.26° (95% CI: 6.24° to 12.28°). Mild asymmetry is visible, indicating a possible but less pronounced publication bias.

**Supplementary Figure S4:** Funnel plot of mean preoperative pelvic tilt (PT) in the supine position. Mean preoperative PT: 5.10° (95% CI: 0.93° to 9.26°). The plot is relatively symmetrical, with minimal signs of publication bias.

**Supplementary Figure S5:** Funnel plot of mean postoperative pelvic tilt (PT) in the sitting position. Mean postoperative PT: 14.64° (95% CI: –52.32° to 81.59°). Asymmetry is visible, suggesting potential small-study effects.

**Supplementary Figure S6:** Funnel plot of mean postoperative pelvic tilt (PT) in the standing position. Mean postoperative PT: 8.49° (95% CI: 4.96° to 12.01°). Mild asymmetry observed.

**Supplementary Figure S7:** Funnel plot of mean postoperative pelvic tilt (PT) in the supine position. Mean postoperative PT: 2.76° (95% CI: –13.12° to 18.64°). Plot appears relatively symmetrical.

**Supplementary Figure S8:** Funnel plot of mean pelvic tilt (PT) difference in the sitting position (postoperative minus preoperative). Mean PT difference: 12.01° (95% CI: –45.28° to 69.30°). Substantial asymmetry suggests possible reporting bias.

**Supplementary Figure S9:** Funnel plot of mean pelvic tilt (PT) difference in the standing position. Mean PT difference: 1.60° (95% CI: –0.57° to 3.76°). Mild asymmetry observed.

**Supplementary Figure S10:** Funnel plot of mean pelvic tilt (PT) difference in the supine position. Mean PT difference: –0.37° (95% CI: –5.44° to 4.70°). The plot is largely symmetrical.

**Supplementary Figure S11:** Forest plot of the mean preoperative pelvic tilt (PT) in the sitting position. The pooled mean PT was 18.14° (95% CI: –16.24° to 52.53°). *CI: confidence interval; SD: standard deviation.*

**Supplementary Figure S12:** Forest plot of the mean preoperative PT in the standing position. The pooled mean PT was 9.26° (95% CI: 6.24° to 12.28°). *CI: confidence interval; SD: standard deviation.*

**Supplementary Figure S13:** Forest plot of the mean preoperative PT in the supine position. The pooled mean PT was 5.10° (95% CI: 0.93° to 9.26°). *CI: confidence interval; SD: standard deviation.*

**Supplementary Figure S14:** Forest plot of the mean postoperative PT in the sitting position. The pooled mean PT was 14.64° (95% CI: –52.32° to 81.59°). *CI: confidence interval; SD: standard deviation.*

**Supplementary Figure S15:** Forest plot of the mean postoperative PT in the standing position. The pooled mean PT was 8.49° (95% CI: 4.96° to 12.01°). *CI: confidence interval; SD: standard deviation.*

**Supplementary Figure S16:** Forest plot of the mean postoperative PT in the supine position. The pooled mean PT was 2.76° (95% CI: –13.12° to 18.64°). *CI: confidence interval; SD: standard deviation.*

**Supplementary Figure S17:** Forest plot of the mean PT difference (postoperative minus preoperative) in the sitting position. The pooled mean difference was 12.01° (95% CI: –45.28° to 69.30°). *CI: confidence interval; SD: standard deviation*.

**Supplementary Figure S18:** Forest plot of the mean PT difference in the standing position. The pooled mean difference was 1.60° (95% CI: –0.57° to 3.76°). *CI: confidence interval; SD: standard deviation.*

**Supplementary Figure S19:** Forest plot of the mean PT difference in the supine position. The pooled mean difference was –0.37° (95% CI: –5.44° to 4.70°). *CI: confidence interval; SD: standard deviation.*

**Supplementary Figure S20:** Forest plot of preoperative PT for the subgroup analysis by operative indication (DDH/BDDH vs. other indications). No statistically significant subgroup differences were observed. *CI: confidence interval; SD: standard deviation.*

**Supplementary Figure S21:** Forest plot of preoperative pelvic tilt (PT) in the sitting position, stratified by operative indication (DDH/BDDH vs. other indications). No statistically significant difference in PT was observed between groups. *PT: pelvic tilt; DDH: developmental dysplasia of the hip; BDDH: borderline DDH; CI: confidence interval; SD: standard deviation.*

**Supplementary Figure S22:** Forest plot of preoperative PT in the standing position by operative indication (DDH/BDDH vs. other indications). No statistically significant differences between indications. *PT: pelvic tilt; DDH: developmental dysplasia of the hip; BDDH: borderline DDH; CI: confidence interval; SD: standard deviation.*

**Supplementary Figure S23:** Forest plot of preoperative PT in the supine position by operative indication (DDH/BDDH vs. other indications). Differences between groups were not statistically significant. *PT: pelvic tilt; DDH: developmental dysplasia of the hip; BDDH: borderline DDH; CI: confidence interval; SD: standard deviation.*

**Supplementary Figure S24:** Forest plot of postoperative PT (overall values) stratified by operative indication (DDH/BDDH vs. other indications). No significant differences detected. *PT: pelvic tilt; DDH: developmental dysplasia of the hip; BDDH: borderline DDH; CI: confidence interval; SD: standard deviation.*

**Supplementary Figure S25:** Forest plot of postoperative PT in the supine position by operative indication (DDH/BDDH vs. other indications). No statistically significant group differences observed. *PT: pelvic tilt; DDH: developmental dysplasia of the hip; BDDH: borderline DDH; CI: confidence interval; SD: standard deviation.*

**Supplementary Figure S26:** Forest plot of overall PT difference (pre- vs. postoperative) stratified by operative indication (DDH/BDDH vs. other indications). No significant differences identified between groups. *PT: pelvic tilt; DDH: developmental dysplasia of the hip; BDDH: borderline DDH; CI: confidence interval; SD: standard deviation.*

**Supplementary Figure S27:** Forest plot of PT difference in the supine position (pre- vs. postoperative) by operative indication (DDH/BDDH vs. other indications). Differences were not statistically significant. *PT: pelvic tilt; DDH: developmental dysplasia of the hip; BDDH: borderline DDH; CI: confidence interval; SD: standard deviation.*

**Supplementary Figure S28:** Forest plot of overall preoperative PT stratified by surgical laterality (unilateral vs. bilateral PAO). No statistically significant differences observed. *PT: pelvic tilt; PAO: periacetabular osteotomy; CI: confidence interval; SD: standard deviation.*

**Supplementary Figure S29:** Forest plot of preoperative PT in the sitting position by surgical laterality (unilateral vs. bilateral PAO). No significant differences detected. *PT: pelvic tilt; PAO: periacetabular osteotomy; CI: confidence interval; SD: standard deviation.*

**Supplementary Figure S30:** Forest plot of preoperative PT in the standing position stratified by surgical laterality (unilateral vs. bilateral PAO). No statistically significant differences observed. *PT: pelvic tilt; PAO: periacetabular osteotomy; CI: confidence interval; SD: standard deviation.*

**Supplementary Figure S31:** Forest plot of postoperative pelvic tilt (PT, overall values) stratified by surgical laterality (unilateral vs. bilateral PAO). No statistically significant differences were detected. *PT: pelvic tilt; PAO: periacetabular osteotomy; CI: confidence interval; SD: standard deviation.*

**Supplementary Figure S32:** Forest plot of postoperative PT in the sitting position by surgical laterality (unilateral vs. bilateral PAO). No significant differences observed. *PT: pelvic tilt; PAO: periacetabular osteotomy; CI: confidence interval; SD: standard deviation.*

**Supplementary Figure S33:** Forest plot of postoperative PT in the standing position stratified by surgical laterality (unilateral vs. bilateral PAO). No statistically significant differences observed. *PT: pelvic tilt; PAO: periacetabular osteotomy; CI: confidence interval; SD: standard deviation.*

**Supplementary Figure S34:** Forest plot of PT difference (pre- vs. postoperative, overall) stratified by surgical laterality (unilateral vs. bilateral PAO). No statistically significant differences identified. *PT: pelvic tilt; PAO: periacetabular osteotomy; CI: confidence interval; SD: standard deviation.*

**Supplementary Figure S35:** Forest plot of PT difference in the sitting position (pre- vs. postoperative) by surgical laterality (unilateral vs. bilateral PAO). No significant differences detected. *PT: pelvic tilt; PAO: periacetabular osteotomy; CI: confidence interval; SD: standard deviation.*

**Supplementary Figure S36:** Forest plot of PT difference in the standing position (pre- vs. postoperative) stratified by surgical laterality (unilateral vs. bilateral PAO). No statistically significant differences observed. *PT: pelvic tilt; PAO: periacetabular osteotomy; CI: confidence interval; SD: standard deviation.*

**Supplementary Figure S37:** Bubble plot showing the association between patient age and preoperative PT (overall values). No statistically significant association was identified. *PT: pelvic tilt; BMI: body mass index.*

**Supplementary Figure S38:** Bubble plot showing the association between male sex and preoperative PT (overall values). No statistically significant association was observed. *PT: pelvic tilt; BMI: body mass index.*

**Supplementary Figure S39:** Bubble plot showing the association between BMI and preoperative PT (overall values). No statistically significant association was detected. *PT: pelvic tilt; BMI: body mass index.*

**Supplementary Figure S40:** Bubble plot showing the association between patient age and preoperative PT in the sitting position. No statistically significant association was found. *PT: pelvic tilt; BMI: body mass index.*

**Supplementary Figure S41:** Bubble plot showing the association between male sex and preoperative pelvic tilt (PT) in the sitting position. No statistically significant association was detected. *PT: pelvic tilt; BMI: body mass index.*

**Supplementary Figure S42:** Bubble plot showing the association between body mass index (BMI) and preoperative PT in the sitting position. No statistically significant association was found. *PT: pelvic tilt; BMI: body mass index.*

**Supplementary Figure S43:** Bubble plot showing the association between patient age and preoperative PT in the standing position. No statistically significant association was observed. PT: pelvic tilt; BMI: body mass index.

**Supplementary Figure S44:** Bubble plot showing the association between male sex and preoperative PT in the standing position. No statistically significant association was detected. *PT: pelvic tilt; BMI: body mass index.*

**Supplementary Figure S45:** Bubble plot showing the association between BMI and preoperative PT in the standing position. No statistically significant association was identified. *PT: pelvic tilt; BMI: body mass index.*

**Supplementary Figure S46:** Bubble plot showing the association between patient age and preoperative PT in the supine position. No statistically significant association was observed. *PT: pelvic tilt; BMI: body mass index.*

**Supplementary Figure S47:** Bubble plot showing the association between male sex and preoperative PT in the supine position. No statistically significant association was found. *PT: pelvic tilt; BMI: body mass index.*

**Supplementary Figure S48:** Bubble plot showing the association between patient age and postoperative PT (overall values). No statistically significant association was detected. *PT: pelvic tilt; BMI: body mass index.*

**Supplementary Figure S49:** Bubble plot showing the association between male sex and postoperative PT (overall values). No statistically significant association was identified. *PT: pelvic tilt; BMI: body mass index.*

**Supplementary Figure S50:** Bubble plot showing the association between BMI and postoperative PT (overall values). No statistically significant association was found. *PT: pelvic tilt; BMI: body mass index.*

**Supplementary Figure S51:** Bubble plot showing the association between patient age and postoperative pelvic tilt (PT) in the sitting position. A statistically significant negative association was observed (β = –4.45; 95% CI: –8.48 to –0.41; p = 0.0454), indicating that postoperative PT decreased with increasing age. *PT: pelvic tilt; BMI: body mass index.*

**Supplementary Figure S52:** Bubble plot showing the association between male sex and postoperative PT in the sitting position. No statistically significant association was found. *PT: pelvic tilt; BMI: body mass index.*

**Supplementary Figure S53:** Bubble plot showing the association between patient age and postoperative PT in the standing position. No statistically significant association was detected. *PT: pelvic tilt; BMI: body mass index.*

**Supplementary Figure S54:** Bubble plot showing the association between male sex and postoperative PT in the standing position. No statistically significant association was observed. *PT: pelvic tilt; BMI: body mass index.*

**Supplementary Figure S55:** Bubble plot showing the association between body mass index (BMI) and postoperative PT in the standing position. No statistically significant association was identified. *PT: pelvic tilt; BMI: body mass index.*

**Supplementary Figure S56:** Bubble plot showing the association between patient age and the overall change in PT (difference between preoperative and postoperative values). No statistically significant association was found. *PT: pelvic tilt; BMI: body mass index.*

**Supplementary Figure S57:** Bubble plot showing the association between male sex and the overall change in PT. No statistically significant association was detected. *PT: pelvic tilt; BMI: body mass index.*

**Supplementary Figure S58:** Bubble plot showing the association between BMI and the overall change in PT. No statistically significant association was found. *PT: pelvic tilt; BMI: body mass index.*

**Supplementary Figure S59:** Bubble plot showing the association between patient age and the change in PT in the sitting position. No statistically significant association was observed. *PT: pelvic tilt; BMI: body mass index.*

**Supplementary Figure S60:** Bubble plot showing the association between male sex and the change in PT in the sitting position. No statistically significant association was identified. *PT: pelvic tilt; BMI: body mass index.*

**Supplementary Figure S61:** Bubble plot showing the association between patient age and the change in PT in the standing position. No statistically significant association was found. *PT: pelvic tilt; BMI: body mass index.*

**Supplementary Figure S62:** Bubble plot showing the association between male sex and the change in PT in the standing position. No statistically significant association was detected. *PT: pelvic tilt; BMI: body mass index.*

**Supplementary Figure S63:** Bubble plot showing the association between BMI and the change in PT in the standing position. No statistically significant association was found. *PT: pelvic tilt; BMI: body mass index.*
